# Retinal genes are differentially expressed in areas of primary versus secondary degeneration following partial optic nerve injury

**DOI:** 10.1371/journal.pone.0192348

**Published:** 2018-02-09

**Authors:** Wissam Chiha, Chrisna J. LeVaillant, Carole A. Bartlett, Alex W. Hewitt, Phillip E. Melton, Melinda Fitzgerald, Alan R. Harvey

**Affiliations:** 1 Experimental and Regenerative Neurosciences, The University of Western Australia, Crawley, WA, Australia; 2 School of Human Sciences, The University of Western Australia, Crawley, WA, Australia; 3 School of Biological Sciences, The University of Western Australia, Crawley, WA, Australia; 4 Lions Eye Institute, The University of Western Australia, Nedlands, WA, Australia; 5 Curtin/UWA Centre for Genetic Origins of Health and Disease, School of Biomedical Science, The University of Western Australia and Curtin University, Bentley, WA, Australia; 6 Perron Institute for Neurological and Translational Science, QEII Medical Centre, Nedlands, WA, Australia; 7 Curtin Health Innovation Research Institute, Curtin University, Bentley, WA, Australia; Universidade Federal do Rio de Janeiro, BRAZIL

## Abstract

**Background:**

Partial transection (PT) of the optic nerve is an established experimental model of secondary degeneration in the central nervous system. After a dorsal transection, retinal ganglion cells (RGCs) with axons in ventral optic nerve are intact but vulnerable to secondary degeneration, whereas RGCs in dorsal retina with dorsal axons are affected by primary and secondary injuries. Using microarray, we quantified gene expression changes in dorsal and ventral retina at 1 and 7 days post PT, to characterize pathogenic pathways linked to primary and secondary degeneration.

**Results:**

In comparison to uninjured retina *Cryba1*, *Cryba2* and *Crygs*, were significantly downregulated in injured dorsal retina at days 1 and 7. While *Ecel1*, *Timp1*, *Mt2A* and *CD74*, which are associated with reducing excitotoxicity, oxidative stress and inflammation, were significantly upregulated. Genes associated with oxygen binding pathways, immune responses, cytokine receptor activity and apoptosis were enriched in dorsal retina at day 1 after PT. Oxygen binding and apoptosis remained enriched at day 7, as were pathways involved in extracellular matrix modification. Fewer changes were observed in ventral retina at day 1 after PT, most associated with the regulation of protein homodimerization activity. By day 7, apoptosis, matrix organization and signal transduction pathways were enriched. Discriminant analysis was also performed for specific functional gene groups to compare expression intensities at each time point. Altered expression of selected genes (*ATF3*, *GFAP*, *Ecel1*, *TIMP1*, *Tp53*) and proteins (GFAP, ECEL1 and ATF3) were semi-quantitatively assessed by qRT-PCR and immunohistochemistry respectively.

**Conclusion:**

There was an acute and complex primary injury response in dorsal retina indicative of a dynamic interaction between neuroprotective and neurodegenerative events; ventral retina vulnerable to secondary degeneration showed a delayed injury response. Both primary and secondary injury resulted in the upregulation of numerous genes linked to RGC death, but differences in the nature of these changes strongly suggest that death occurred via different molecular mechanisms.

## Introduction

Traumatic injury to the central nervous system (CNS) is the direct damage of brain or spinal cord tissue by a physical insult. Trauma also disrupts the physiological activity of homeostatic systems and auto-regulation of blood flow to intact regions, and triggers diverse inflammatory responses that together create a toxic environment conducive to delayed, secondary degeneration of tissue not initially affected by the trauma. This secondary degeneration results in more widespread pathology and even greater functional loss. Evidence suggests that secondary degeneration is a common factor following trauma to the brain [1] and spinal cord [2], after stroke [3], and in various neurodegenerative diseases including, for example, glaucoma [4].

An understanding of the degenerative sequelae in neurons and adjacent glia following an initial injury is enhanced by being able to distinguish between neural tissue containing directly axotomized neurons and tissue containing intact, but subsequently vulnerable, neurons. Partial transection (PT) of the dorsal aspect of the rat optic nerve (ON) in Piebald Viral Glaxo (PVG) rats is now an established CNS model to simultaneously study a primary injury response and secondary degeneration following trauma [5–8]. This is because retinal ganglion cell (RGC) bodies located in ventral retina are not axotomized by the primary injury but are vulnerable to secondary degeneration, while RGCs in dorsal retina are affected by both the primary and secondary injuries [9]. The model allows crucial topographic/spatial separation of primary versus secondary degenerative events.

Secondary degeneration is characterized by electrolytic shifts and increases in extracellular concentrations of glutamate and other excitatory amino acids, which reach cytotoxic concentrations as a result of cell lysis from mechanical injury and glial release [10–12]. Mitochondrial dysfunction has also been implicated, associated with mitochondrial Ca^2+^ overloading and increased reactive species [13–16]. This leads to the opening of mitochondrial transition pores, loss of mitochondrial membrane potential, ATP depletion, cytochrome *c* release and increases in oxidative stress, associated with further release of Ca^2+^, mitochondrial swelling and lipid peroxidation [15, 17–19]. Secondary degeneration often culminates in cell death, predominantly *via* apoptotic and to some extent necrotic mechanisms [6, 9, 20, 21].

Biochemical changes following neurotrauma are increasingly well characterized, however fewer studies have attempted a direct comparison of the genetic changes in CNS regions vulnerable to primary compared to secondary degeneration. One report using the optic nerve PT model identified significant down-regulation of pro-survival genes Bcl-2 and Bcl-x-L and up-regulation of pro-apoptotic genes Bax, Bad and inhibitor of apoptosis protein-1 (IAP-1)[22]. Secondary degeneration was characterized by a delay in the up-regulation of Bax and Bad [22]. Growth arrest and DNA damage inducible protein 45α (GADD45α), cyclin-dependent kinase 2 (CDK2) and etoposide-induced protein 2.4 homolog (ei24) activation in the retina were also associated with both primary and secondary degeneration [23]. These results suggest potential mechanistic similarities but temporal variations between the progression of primary and secondary neurodegenerative events.

Microarray based analysis of the transcriptomic changes in the retina following PT of the ON can potentially provide important new and additional insights into the mechanisms and pathways involved in primary and secondary degeneration in CNS tissues. The interpretation of microarray data is complicated by several features. Firstly, the reliability of data depends on using a sufficient number of animals to provide biological replicates. Secondly, the large body of data generated requires appropriate normalization and multiple analysis methods to allow the separation of biologically relevant from incidental changes. Additionally, in whole retina expression studies, outcomes reflect contributions from many cell types including neurons, glia and vasculature, not just RGCs. Nonetheless, such an analysis yields important information about the overall tissue response to the experimental perturbation, which in many ways is more relevant to the design of protective therapies. Here, we quantified changes in gene expression of dorsal and ventral retina at 1 and 7 days post PT, to identify genetic changes associated with primary and secondary injury. Many novel changes in gene expression were seen, with a rapid injury response that was almost entirely confined to dorsal retina containing neurons directly affected by the injury. Ventral retinal tissue containing RGCs affected by secondary degeneration showed a delayed response to injury. A direct comparison between dorsal and ventral retina at days 1 and 7 post injury suggests that initially after injury, there are robust gene expression differences, which was associated with proliferative functional pathways. As the degenerative sequel progresses, gene expression profile remains different at day 7, however, these genes are associated with fewer and different functional groups, strongly suggestive of differential degenerative events in regions of retina vulnerable to primary versus secondary injury.

## Experimental procedures

### Animals

PVG rats (160–190 g) were obtained from the Animal Resource Centre (Murdoch W.A.) and housed in clear plastic cages with food and water *ad libitum* and subjected to a standard 12-hour light / dark cycle. All experimental procedures conformed to ‘Principles of Laboratory Animal Care’ and were approved by the Animal Ethics Committee of The University of Western Australia (approval number RA3/100/673). Animals were euthanized with Euthal (active constituents Pentobarbitone Sodium 170 mg/ml, Phenytoin Sodium 25 mg/ml) at days 1 or 7 post-surgery; uninjured control animals were euthanized in the same way.

### Partial transection of the optic nerve and retinal dissection

**Fig 1:**
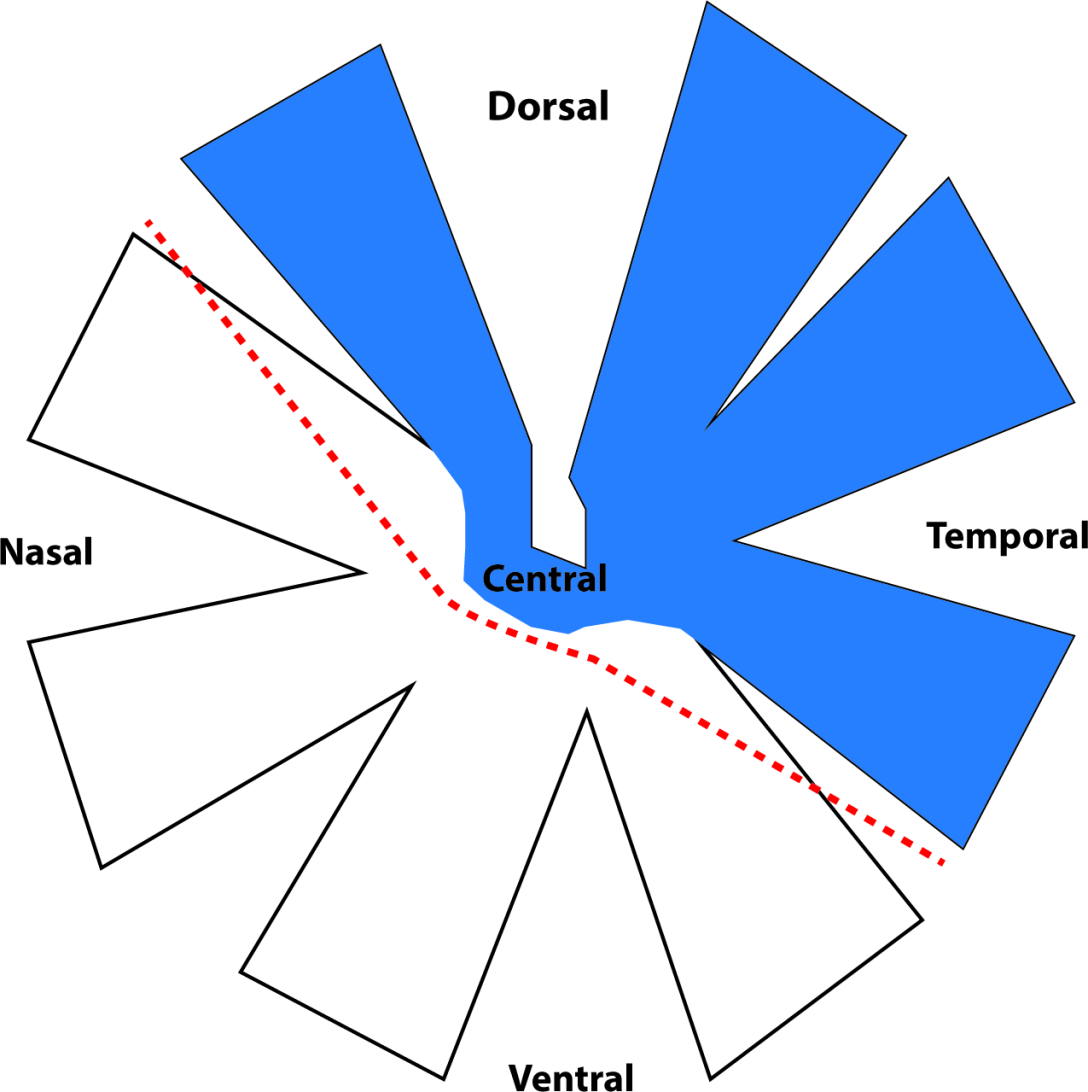
Schematic diagram of dissection of retina for microarray studies. RGC somata whose axons are transected after PT of the optic nerve are located in the dorsal, temporal and central areas (indicated in blue), leaving RGCs in nasal and ventral retina initially intact but vulnerable to secondary degeneration[26]. The dashed line indicates where dorsal and ventral retinal tissue were separated.

Unilateral PT of the ON was performed as previously described [5, 6, 9]. Briefly, PVG rats were anaesthetized with ketamine (50 mg/kg)/ xylazine (10 mg/kg, Troy Laboratories, NSW, Australia) administered intraperitoneally. An incision was made in the skin overlying the right eye and access to orbit was made with blunt dissection. Lachrymal glands were deflected and extra ocular muscle incised to access the nerve. A 200-μm incision was made on the dorsum of the optic nerve approximately 1 mm behind the optic nerve head using a diamond keratotomy knife (Geuder, Germany). Post-operative analgesia was administered subcutaneously (2.8 mg/kg carprofen, Norbrook Australia, Pty. Ltd., VIC, Australia) and animals recovered on a warming blanket. The contralateral ONs were not injured and were not used as controls due to demonstrated bilateral changes following unilateral ON injury [24, 25]. Uninjured, age-matched animals were used as controls, as we have previously demonstrated no significant differences between sham-operated and normal animals in relevant outcomes including RGC numbers [26.]. The eyes were dissected (cornea and lens removed) and orientation was maintained using a dorsal incision and retinae cut into two halves (Fig 1), thereby separating primarily dorsal retina containing RGCs affected by the primary injury from more ventral retinal regions exclusively vulnerable to secondary degeneration [9]. Retinae from uninjured control animals were similarly partitioned. The retinal halves were immersed in RNA later (Qiagen) and stored at -80°C.

### RNA extraction

Retinal halves from each animal were homogenized separately with a Kontes Pellet Pestle mechanical homogenizer (2000–3000 RPM) until tissue lysates were finely dispersed. Tubes and the homogenizer were rinsed with DEPC water or RNA zap (Dimethyl dicarbonate, 1:1000, Sigma-Aldrich) respectively prior to use, to inactivate RNase. RNA was extracted in accordance with the RNeasy MINI extraction protocol (RNeasy Mini Kit, Qiagen). The quality and quantity of total RNA was determined for each preparation (Bioanalyzer; Agilent). Typical of retinal studies [27, 28] three biological replicates were used for each experimental group, with 600 ng of total RNA from each retinal half analyzed.

### Microarray analysis and data analysis

The mRNA samples from normal and injured animals at days 1 and 7 were assessed using Affymetrix GeneChip Gene 2.0 ST Array. RNA labelling hybridization, staining, and scanning were performed by The Ramaciotti Centre for Gene Function Analysis, (Sydney, Australia). Experiment normalization and statistical analysis were performed using R (3.2.2 version). We used the Robust Multichip Average (RMA) model for array background correction and quantile normalisation (REF: PMID: 12538238; 12582260; 12925520). Probes were mapped to the Rattus norvegicus genome (RGSC 5.0/rn5). Results from dorsal and ventral retinal samples at day 1 and 7 post injury were compared to the results from dorsal and ventral retinal arrays from normal uninjured animals, to allow the identification of primary and secondary degeneration regulated sequences. A direct comparison of gene expression changes was also made between dorsal and ventral retina at 1 and 7 days after ON injury. Differentially regulated genes were extracted using the R statistical package [29] with a cut-off for these sequences at a p ≤ 0.05 and a minimum regulation > (±) log_2_0.5 Fold change (FC) ~ 1.4 (FC).

### Enrichment analysis of differentially expressed genes

Gene set enrichment analysis (GSEA) of the differentially expressed genes was conducted using Pathway Studio Mammal (Elsevier, Netherlands). GSEA was used to identify statistically significant enrichment in genes of known functional groups and curated pathways, considering their expression intensity. Gene set categories were set to the following Gene Ontology (GO) categories: ‘molecular_function’, ‘cellular_component’ ‘biological_process’ and ‘Pathway studio ontology’. An enrichment p-value ≤ 0.05 was applied in order to filter GSEA output. Functional pathways were supported by data extracted from the literature using MedScan and added in ResNet databases, which can be accessed through the *Pathway* studio interface.

Genes in the clusters associated with oxidative stress, immune response and apoptosis were further analyzed using PANTHER Gene Ontology to identify sub clusters [30]. Discriminant analysis was performed on the expression intensity of genes contained within each sub cluster to compare the expression intensities across the different retinal tissues, to determine whether a set of variables was effective in predicting category membership of a particular retinal tissue group.

### Confirmatory RT_PCR

The same RNA used in the microarray analysis was reverse transcribed to cDNA (QuantiTect kit) using 500 ng template RNA per reaction, for confirmatory PCR. RT-PCR reactions were performed using 5 μl IQ PCR mix (BioRad, Australia) with 1μl of total template cDNA from each sample, 1μl of each primer diluted to a 5 μM working solution (Table 1) and 1μl sterile water to a final volume of 10μl. qPCRs were performed with a RotorGene RG 6000 (Qiagen, USA). Three reference or ‘housekeeper’ genes were tested; PPIA, HPRT1, TBP, and were selected for all analyses after confirmation. For each gene tested, all experimental and control retina were assessed in the same experiment, n = 3 were used and data pooled from two experimental runs. The data were normalized to the geometric mean of the three housekeeper genes and expressed as arbitrary units of expression.

**Table 1:** Primers used for qRT-PCR array validation.

Gene	Gene name	Accession number	Temp	size (bp)	Primer
**Atf3**	activating transcription factor 3	NM_012912	60°C	233	F: CCAGAACAAGCACCTTTGCC R: CGGCATTCACACTCTCCAGT
**Ecel1**	Endothelin converting enzyme-like 1	NM_021776	60°C	134	F: TCTCGACATGCGTGAGATCG R: ACAACAGTCGGTTGAGGTCC
**GFAP**	glial fibrillary acidic protein	NM_017009	60°C	143	F: GAAATTGCTGGAGGGCGAAG R: TCTCCACCGTCTTTACCACG
**Timp1**	TIMP metallopeptidase inhibitor 1	NM_053819	60°C	140	F: CGCTAGAGCAGATACCACGA R: CCAGGTCCGAGTTGCAGAAA
**Tp53**	Tumor protein p53	NM_030989	60°C	217	F: CTGGACGACAGGCAGACTTT R: GACAGGCACAAACACGAACC

### Immunofluorescence and microscopy

Animals were sacrificed at days 1 and 7 post injury and perfused transcardially with saline followed by 4% paraformaldehyde (ACROS ORGANICS MS); uninjured control animals were processed similarly (N = 6 / group). The eyes were post-fixed in 4% paraformaldehyde overnight, cryoprotected by immersion in 15% sucrose in PBS, before they were embedded in optimal cutting temperature (OCT) compound (Sakura Finetek USA) and cryosectioned (20μm) along the dorsal/ventral axis. If not processed immediately, the slides were stored at -80°C. Retinal sections were air dried, rehydrated in PBS, and incubated overnight at 4°C in primary antibodies diluted in PBS+ 0.2% Triton X100. Primary antibodies were rabbit ATF-3 (1:250 dilution; Santa Cruz Biotechnology); rabbit GFAP (1:500; Sigma); mouse β-III tubulin (1:500; Covance), goat Ecel1 (1:500, R&D Systems) and goat Brn3A (1:500, Santa Cruz Biotechnology). Antibody binding was visualized following 2-hour incubation with appropriate Alexa Fluor 488, 555 and 647 secondary antibodies (1:400; Molecular Probes). Slides were washed in PBS and cover-slipped using Fluoromount-G (Southern Biotechnology).

For semi-quantification of immunofluorescence intensity in retinal sections, the dorsal, central and ventral areas were visualized and photographed using a Nikon Eclipse Ti inverted microscope (Nikon Corporation, Japan) or confocal Nikon c2 mounted on an upright Ni-E microscope, controlled by NIS elements 4.3 software at 20x magnification. A series of optical images at 0.5μm increments along the z-axis was acquired from the middle 6μm of each 20μm section. Images collected on the Nikon Eclipse Ti inverted microscope were deconvoluted using a custom-made Macro (design: Nathaniel Yates) using the AQI Deconvolution ND function and batch processing feature in NIS Elements. All images for each outcome measure were captured at constant exposures and in a single session. Image analysis was conducted on Image J/Fiji analysis software, setting constant arbitrary threshold intensities for each image, and semi-quantifying mean intensities and areas above the set threshold. Immunointensity data were normalised to background within the same section to adjust for variations in section thickness and staining application. Statistical analyses were performed using ANOVA followed by Bonferroni multiple comparison *post hoc* tests, requiring a significance value of p ≤ 0.05.

## Results

### Gene expression analysis of retinal tissue 1 and 7 days post PT injury

Whole genome microarray technology was used to profile gene expression changes in dorsal and ventral retina following PT injury, relative to control retina. Microarray analysis identified 2220 genes that were differentially expressed (1.4 ≤ FC ≤ -1.4, p ≤ 0.05) in the dorsal retina directly affected by the primary injury at day 1, with a similar proportion of upregulated versus downregulated genes. Expression of 238 genes was downregulated by 2-fold or more and 30 of these genes were downregulated by 3-fold or more at day 1 (Table 2). Expression of 146 genes increased more than 2-fold and 19 genes were upregulated by 3-fold or more at day 1 following injury (Table 3). At day 7 following injury, the number of genes differentially expressed (p ≤ 0.05) in dorsal retina increased to 2330 and of these, 968 genes were upregulated. The expression of 17 genes was upregulated by 2-fold or more, of which 11 genes were upregulated by at least 3-fold, while the expression of 10 genes was downregulated by at least 2-fold, of which 4 were downregulated by at least 3-fold.

**Table 2:** Downregulated genes in dorsal and ventral retina following a PT injury. Fold changes comparing injured retina to region matched normal controls.

Gene Symbol	GeneBank ID	Dorsal retina / FC Injured/normal		Ventral retina/ FC Injured/normal		Function
		Day 1	Day 7	Day 1	Day 7	
***Cryba1***	NM_013056	-11.30 *	-16.10*	-3.80		Constituents of eye lens
***Cryba2***	NM_173140	-6.95	-10.85 *	-3.50		Constituents of eye lens
***Crygs***	NM_173140	-5.05	-7.50 *	-3.05		Constituents of eye lens
***Ndrg4***	NM_031967	-3.20 *	-2.34*			Positive regulation of ERK1 and ERK2 signaling pathway
***Slc6a13***	NM_133623		-3.63 *			Neurotransmitter activity (Sodium symporter)
***Cxcr6***	NM_001102587	-2.70*		1.6		Chemokine receptor
***N5***	NM_022857	-2.70*	-2.10*		1.90	DNA binding protein
***Hbp1***	XM_347266	-2.50*				Transcriptional regulator
***Gif***	NM_017162	-2.40*	-2.40*			Binds vitamin B12
***Krtap1-3***	XM_001055744	-2.40*				Keratin associated protein
***Rhot1***	NM_001107026	-2.00*			1.90*	Mitochondrial outer membrane permeabilization
***Lipogenin***	NM_145790	-2.00*				
***Mip***	NM_001105719		-2.80*			Cellular water homeostasis
***Fam134B***	NM_001034912		-2.20*	1.5*		Long-term survival of nociceptive and autonomic ganglion neurons.
***Ndufb4l1***	XM_001065938		-2.20*			Oxidation-reduction process

Expression of significantly downregulated genes by fold change ≥ 2. Significance of p-value is indicated by *. Values that may not fall within those parameters are added for the purpose of comparison. Identified and known genes included in the table.

**Table 3:** Upregulated genes in dorsal and ventral retina following a PT injury. Fold changes comparing injured retina to region matched normal controls.

Gene Symbol	GeneBank ID	Dorsal retina / FC Injured/normal		Ventral retina/ FC Injured/normal		Function
		Day 1	Day 7	Day 1	Day 7	
***Ecel1***	NM_021776	16.20 *	11.80 *		2.10	Proteolysis
***Timp1***	NM_053819	4.60 *	3.30 *	-2.25 *		Metalloendopeptidase inhibitor activity
***Mt2A***	NM_001137564	4.50 *	4.30 *			Cellular zinc ion homeostasis
***CD74***	NM_013069	4.10 *	4.10 *			Activation of MAPK activity
***Gfap***	NM_017009	4.00 *	3.45 *	-2.20 *		Response to wounding (glia)
***S100a11***	NM_001004095	3.40 *	2.40*			Calcium ion binding
***Gpnmb***	NM_133298	3.40 *	2.80 *			Cell adhesion
***Tmem47***	NM_001109317	3.30 *	2.40 *			Cell-cell junction
***Egr1***	NM_012551	3.30 *	2.80 *			Negative regulation of transcription from RNA polymerase II promoter
***Arhgdib***	NM_001009600	3.20 *	2.50 *			Rho GDP-dissociation inhibitor activity
***H2afy2***	NM_001135807	3.20 *	2.50 *			Nucleosome assembly
***Bgn***	NM_017087	3.10 *	2.40*			Blood vessel remodelling
***Tagln2***	NM_001013127	3.00 *	2.70 *		1.50*	Tumor suppressor
***Dhdh***	XM_003748855	2.90 *				D-xylose catabolic process
***Sprr1a***	NM_021864	2.90 *	2.10 *			Peptide cross-linking
***Atf3***	NM_012912	2.90 *	2.40*	1.50*		Cell fate determination
***Tmem108***	XM_008757857		2.10*		2.20*	
***Rgr***	NM_001107299		2.30*		2.30*	Retinal G-protein coupled receptor

Expression of significantly upregulated genes by fold change ≥ 2. Significance of p-value is indicated by *. Values that may not fall within those parameters are added for the purpose of comparison. Identified and known genes included in the table.

Genes associated with secondary degenerative changes in ventral retina following PT injury were analyzed in comparison to ventral retina of control animals. At day 1 following injury 413 genes were differentially expressed (1.4 ≤ FC ≤ -1.4, p ≤ 0.05) in ventral retina. Expression of 24 genes was altered by at least a 2-fold change, of which only 3 have to date been characterized. Each of these identified genes was downregulated (*GFAP*, *LCN2* and *TIMP1*). The number of differentially expressed genes in ventral retina increased to 626 at day 7 after PT injury. Expression of 58 genes was significantly upregulated by at least 2-fold; of these, only 2 genes are known being integral components of the membrane and play a role in mediating chemokine receptor activity. Of the 30 genes that were downregulated by 2-fold or more, only 3 have been characterized and were: *Tnfrsf11b*, a member of the tumor necrosis factor receptor superfamily, *Terc*, telomerase RNA component, and *Olr1460* (Table 2).

### Spatial and temporal comparison of significantly regulated genes in PT and control retina

The most significantly downregulated genes included members of the crystallin family. Expression of *Cryba1* was increasingly downregulated from -11 FC at day 1 to -16 FC by day 7 in dorsal retina. In ventral retina, *Cryba1*expression was significantly downregulated by -3 FC at day 1, but was not differentially expressed compared to control at day 7. The spatial and temporal expression changes of *Cryba2* and *Crygs* followed the same pattern of expression as *Cryba1*, but to a lesser magnitude and did not reach significance. In contrast, expression of *NDRG4* was downregulated by -3 FC in dorsal retina at day 1 and by -2 fold by day 7; no similar expression changes were seen in ventral retina.

Compared to respective dorsal or ventral control retina, prominent upregulation of several genes (Table 3) was seen in dorsal but not ventral retina at days 1 and 7. The most upregulated gene was endothelin converting enzyme-like 1 (*Ecel1*) with a greater than 16-fold increased expression at day 1 in dorsal retina and an 11-fold increase at day 7. A 2-fold increase in expression of Ecel1 was observed at day 7 in ventral retina, but this did not reach statistical significance. Other neuroprotective genes that were significantly upregulated compared to controls include metallopeptidase inhibitor 1 (*TIMP1*), metallothionein 2A (*MT2A*) and major histocompatibility complex, class II invariant chain (*CD74*). In dorsal retina, the expression of *TIMP1* and *MT2A* was reduced from a 4.6 and 4.5-fold upregulation at day 1 to 3.3 and 4.3-fold respectively at day 7, while the expression of CD74 did not change. In contrast, in ventral retina TIMP1 was downregulated at day 1 by a 2.3 FC. The expression of glial fibrillary acidic protein (*GFAP*) was also upregulated in dorsal retina with 4-fold increased expression at day 1 lessening to a 3.5-fold increased expression at day 7. In ventral retina, differential expression of *GFAP* was seen only at day 1 with a 2.2 fold downregulation. Interestingly, expression of retinal g-protein coupled receptor (RGR) and transmembrane protein 108 (TMEM108) were significantly upregulated at day 7 in both dorsal and ventral retina.

Overall, gene expression data derived from dorsal versus ventral retina were for the most part quantitatively and temporally different, which strongly suggests mechanistic differences in primary and secondary degenerative events.

### Gene set enrichment analysis

GSEA was performed on the list of genes with a cut-off of Log_2_ FC ≥ 0.5 (~FC ≥ 1.4) and p-value ≤ 0.05. The analyses were conducted using *Pathway studio* Ontology and Gene Ontology (GO) and included the descriptors ‘biological processes’, ‘cellular components’ and ‘molecular functions’.

#### Dorsal retina

**Fig 2:**
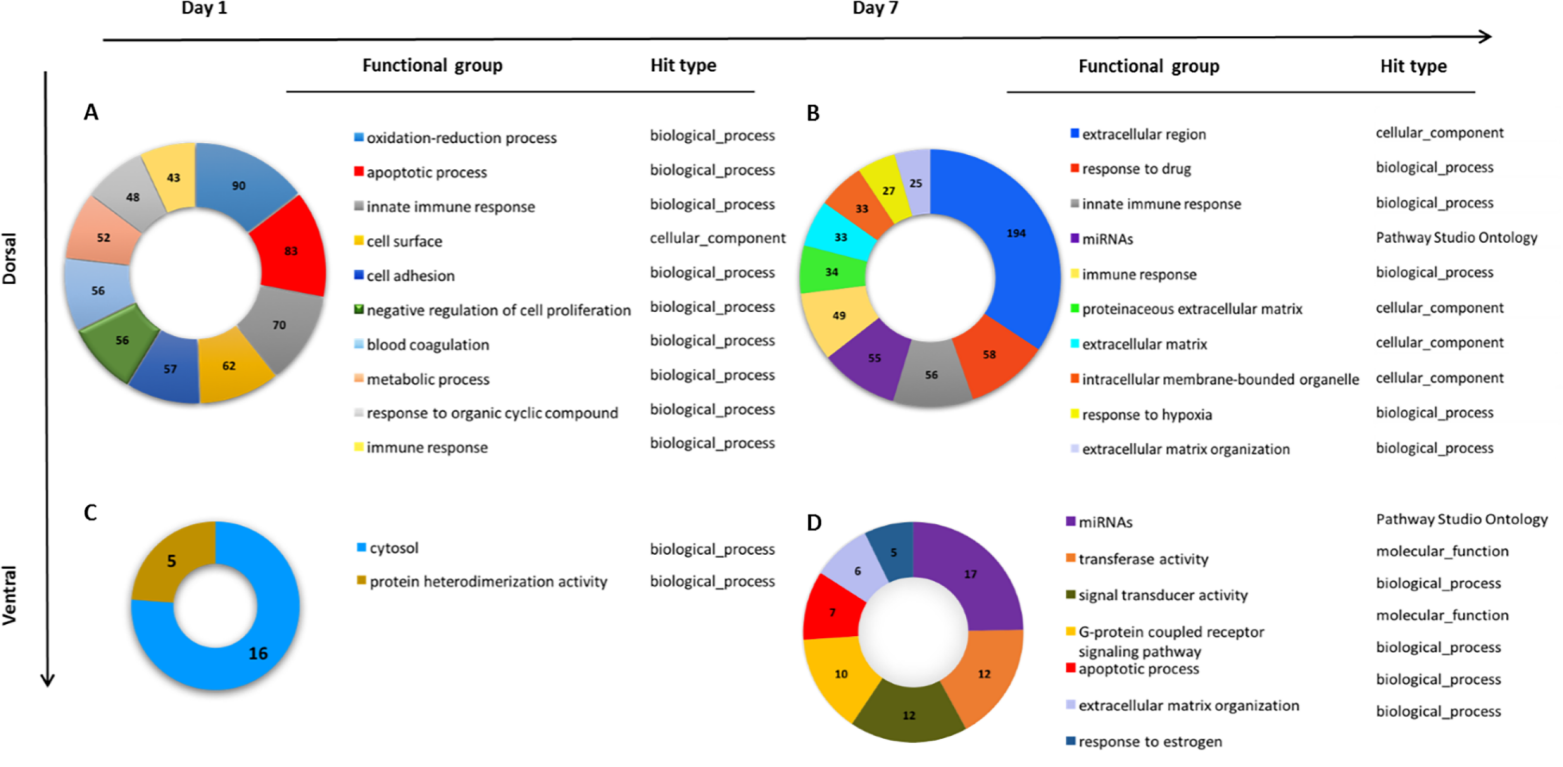
GSEA analysis was performed on a list of significantly differentially expressed genes using a cut off ≥ 1.4 fold change and p-value ≤ 0.05. Pie charts show numbers of genes from experimental data present in each functional category listed in the legend. The order of listing of functional groups is determined by the number of genes present in each category. Hit obtained from *Gene Ontology (GO) categories*: *‘molecular_function’*, *‘cellular_component’ ‘biological_process’ and ‘Pathway studio ontology’* (A) Enriched functional groups in dorsal retina 1 day following PT injury compared to dorsal uninjured retina yielded significant differences in 88 functional groups. The top 10 functional groups listed based on number of genes present in each functional group (B). Enriched functional groups between dorsal retina 7 days following PT injury and dorsal uninjured retina yielded 67 significant functional groups. (C) Enriched functional groups in ventral retina 1 day following PT injury compared to ventral uninjured retina: only 2 significant functional groups were enriched. (D) Enriched functional groups in ventral retina 7 days following PT injury compared to ventral uninjured retina yielded 7 significant functional groups.

The most regulated genetic biological process, based on the number of genes differentially expressed (FC ≥ 1.4 and p = 0.04) in dorsal retina exposed to the primary injury, was the oxidative-reduction process with 90 genes differentially expressed at day 1 (Fig 2A). This was closely followed by apoptotic process enriched pathways with 83 differentially expressed genes (p = 0.0001). Further analysis of the apoptosis pathways differentiated 8 sub- clusters under the heading of cell death, including programmed cell death and positive regulation of extrinsic and intrinsic apoptotic signalling pathways, regulation of mitochondrial permeability and positive regulation of cytochrome c release. Negative regulation of cell proliferation was also significantly enriched with 56 differentially expressed genes (p = 0.0008) (Table A in S1 Text). The immune response also appears to play a large role early in the primary injury with 8 enriched sub-clusters, including activation of the innate and adaptive immune systems as well as enrichment of genes associated with cytokine receptor activity (70 genes present, p = 0.03) (Table A in S1 Text). Sixty-two differentially expressed genes were enriched for ‘cell surface’, under the cellular component descriptor, and 57 genes clustered under ‘cell adhesion’ (Table A in S1 Text).

By day 7 after PT, in dorsal retina the immune response appeared to play a less extensive role with only 4 sub-clusters significantly enriched (Fig 2B, Table B in S1 Text). At this time-point, in dorsal retina affected by the primary injury, the dominant change compared to controls was associated with modifications of the extracellular region. There were 13 sub-clusters of differentially expressed genes, which included genes related to structural constituents and organization of extracellular matrix. Interestingly, glutathione synthesis and activation was also enriched in dorsal retina at day 7, characterized by 9 genes enriched in the glutathione derivative biosynthetic process and 19 genes enriched in glutathione metabolic and peroxidase activity (Table B in S1 Text). Although only two clusters were associated with apoptotic cell death in dorsal retina 7 days post PT, they included positive regulation of the intrinsic apoptotic signalling pathway (5 genes, p = 0.04) and positive regulation of the neuronal apoptotic process (9 genes, p = 0.01). The structural breakdown of the cell was implicated, with enrichment of genes associated with scavenger receptor activity (9 genes, p = 0.04) and protease binding (12 genes, p = 0.01). We also observed 55 differentially genes that are associated with miRNAs (micro-RNA) enriched in dorsal retina 7 days post PT.

#### Ventral retina

Secondary degeneration in tissue from ventral retina was characterized by significant changes in two gene clusters at day 1, one with non-specific functional attributes including cytoplasmic organelles (16 genes, p = 0.01) and the other associated with the formation of protein heterodimers (5 measured genes, p = 0.006, Fig 2C, Table C in S1 Text). By 7 days after PT, we found enrichment of genes associated with apoptotic processes (7 genes, p = 0.04) with 4 genes common with dorsal retina, and the differential expression of *ADAMTSL4*, *BCL2L1* and *BCL2L10* which appears to be unique to ventral retina at day 7 (data not shown). Six gene clusters associated with extracellular matrix organization were enriched, (Fig 2D, Table D in S1 Text, p = 0.01) with *MMP14*, *ADAMTSL4 and MMP19* unique to ventral retina at day 7. Compared to comparable regions of control retina, gene clusters associated with transferase activity, signal transducer activity and G-protein coupled receptor signalling pathway were only enriched in ventral retina at day 7 PT, with 12, 12 and 10 differentially expressed genes respectively. Ventral retina was also enriched with 17 genes that are associated with miRNA 7 days post injury.

### Dorsal versus ventral retinal comparisons in PT animals

Differential gene expression between dorsal and ventral retina identified over 800 significantly differentially expressed genes (~FC >1.4, p ≤ 0.05) at days1 and 7 after partial ON injury. In injured animals, significantly upregulated genes in dorsal retina when compared to ventral retina included Ecel1, *GFAP*, *CD74*, *TIMP1*, *Sprr1A* and *MT2A* (FC > 2.5, p ≤ 0.05), genes that were also upregulated in dorsal retina following injury when compared to normal dorsal retina (Table 3). *Ecel1*, *GFAP*, *CD74* and *MT2A* remained significantly upregulated 7 days post injury, on the other hand *CD244*, and *TNFRSF11B* (FC > 2, p ≤ 0.05) were differentially expressed only at day 7 in dorsal retina when compared to ventral retina. Expression of members of the crystallin family was not significantly different between dorsal and ventral retina following injury at either time point.

**Fig 3:**
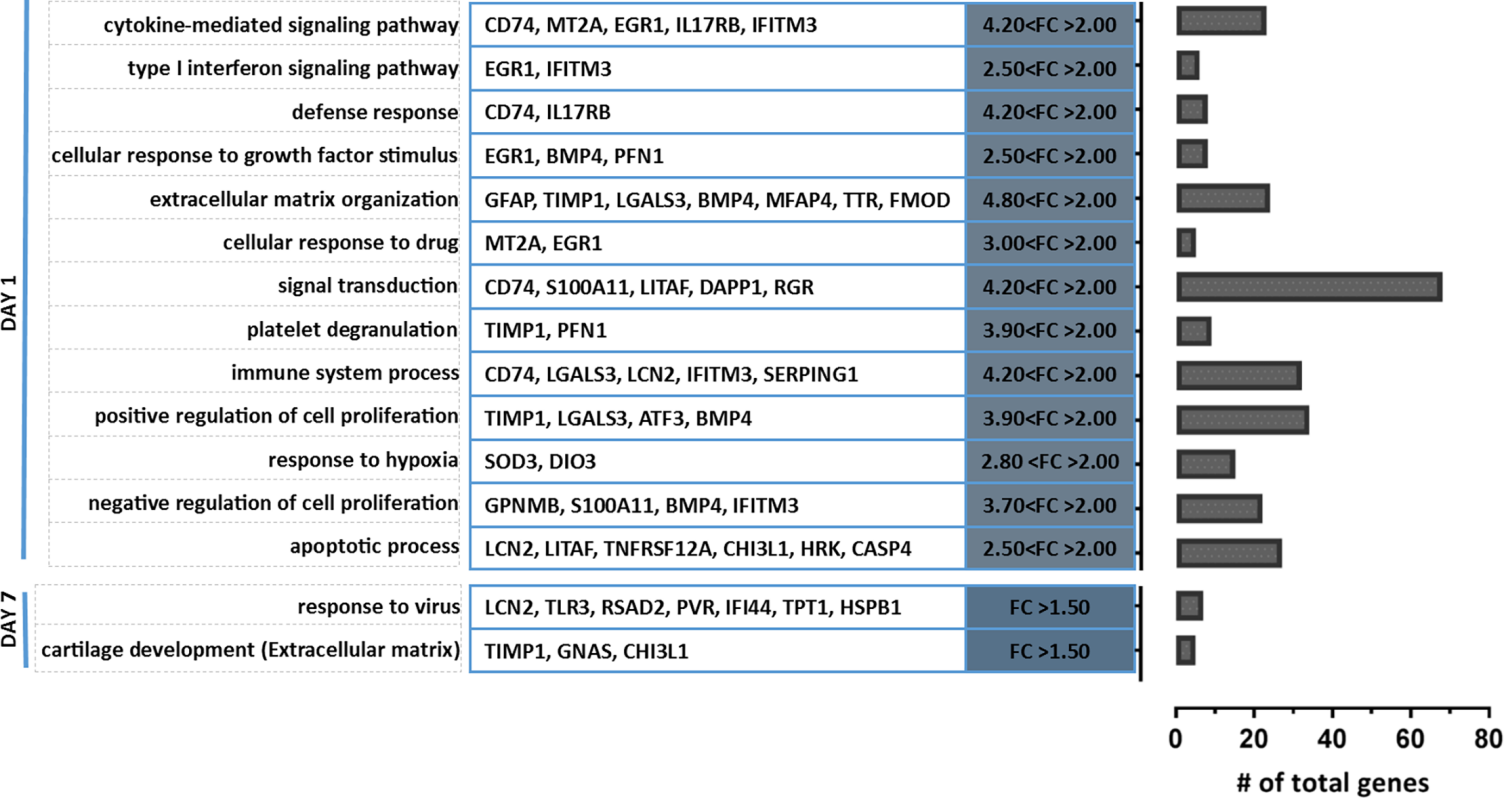
GSEA of differentially expressed genes (FC > 1.4, p-value < 0.05) between dorsal and ventral retina, showing GO classified biological_functions at day 1 and day 7 after PT injury. All biological_function clusters are upregulated in dorsal retina when compared to ventral retina. Top 10 significant (p-value < 0.05) biological_functions shown for day 1 with a list of contributing genes that are differentially expressed by fold change > 2, p-value < 0.05. Only 2 clusters were enriched from differentially expressed genes between dorsal and ventral retina at day 7 after injury. The genes listed are differentially expressed by a FC > 1.5. The bar graph depicts the total number of genes that contribute to each functional cluster.

GSEA was performed on the list of differentially expressed genes between dorsal and ventral retina after ON injury with a cut-off of Log_2_ FC ≥ 0.5 (~FC ≥ 1.4) and p ≤ 0.05. *Pathway Studio* was used to perform the Gene ontology (GO) analysis with a focus on ‘biological process’. A total of 35 biological processes were significantly enriched at day 1 after ON injury, several of which were associated with the inflammatory response. The cytokine mediated signalling pathway was the most significantly enriched biological process (p-value ≤ 0.001, Fig 3). This was followed by type I interferon signalling pathway (p ≤ 0.01) which signals widely expressed cytokines with antiviral and growth inhibitory modalities. Defence response and immune system processes are also inflammation associated pathways which were significantly enriched 1 day after ON injury. Four significantly upregulated genes (FC > 2, p ≤ 0.05) which include EGR1, TIMP1, IFITM3 and CD74, contributed to several biological processes. It is also important to note that the expression profile of apoptosis associated genes was significantly different between dorsal and ventral retina at day 1 after PT. Conversely at day 7 following injury, the expression profile of differentially expressed genes between dorsal and ventral retina revealed only two significantly enriched biological processes, generically described as response to a virus and cartilage development, but comprising a set of genes that are also associated with the extracellular matrix.

### Discriminant analysis on functional groups

Functional groups associated with oxidative stress, immune response and apoptosis were further subdivided using PANTHER Gene Ontology to identify sub clusters. Discriminant analysis was performed on genes within each sub cluster to compare expression intensities across the different retinal tissues to determine whether a set of variables was effective in predicting category membership of a given retinal tissue. Categories were selected for further detailed analysis based on marked changes in individual genes or clusters. Including oxidative stress, which was enriched with 90 genes in dorsal retina at day 1 after PT, genes associated with immune response were common in dorsal retina at day 1 and 7 post PT and genes associated with apoptosis, which were common between dorsal and ventral retina following injury. Additionally our previous studies on changes in proteins in the optic nerve and retina after PT injury also indicated the importance and relevance of oxidative stress, immune responses and events associated with apoptosis in secondary degeneration [6, 16, 26].

#### Oxidative stress

Discriminant analysis of oxidation-reduction process variables demonstrated that following PT injury both dorsal and ventral retina were significantly different from normal control retina at day 1 post injury. Interestingly in the injured retina at day 1 post PT, there was no difference in the expression of oxidation-reduction process associated genes between dorsal and ventral retina. While changes to the oxidation-reduction process remained different at day 7 in dorsal retina following PT when compared to dorsal uninjured retina, ventral retina was indistinguishable from ventral retina of normal control animals (p > 0.05. Table 4A).

**Table 4:** Discriminant analysis on biological clusters related to oxidation-reduction, immune response and apoptosis after partial optic nerve injury.

		D1 PTD-NormD	D1 PTV-NormV	D7 PTD-NormD	D7 PTV-NormV	D7 PTD-D1 PTD	D7 PTV-D1 PTV	D1 PTV-D1 PTD	D7 PTV-D7 PTD
**A**	***Oxidative stress***								
	**Oxidation-reduction process**	**	**	**	**0.99**	**	**	**0.26**	**
	**Glutathione metabolic process**	**	**	**	**	*	**0.28**	**0.68**	**0.99**
**B**	***Immune response***								
	**Adaptive immune response**	**	**	**	**	**	**0.09**	**0.51**	**0.82**
	**Innate immune response**	**	**	**	**	**0.51**	**0.48**	**	**
	**Cytokine-mediated signalling pathway**	**	**	**	**	**0.46**	**	**	**
**C**	***Apoptosis***								
	**Apoptosis**	**	**	**	**	**	**	**	**
	**Extrinsic apoptotic pathway**	**	**	**	**	**	**	**	**
	**Intrinsic apoptotic pathway**	**	**	**	**	**	**	**	**
	**Intrinsic-response to ER stress**	**	**	**	**	**0.2**	**0.94**	**	**0.87**
	**Intrinsic-response to DNA damage**	**	**	**	**	**	**	**	**0.91**
	**Regulation of mitochondrial membrane permeability**	*	**	**	**	**0.2**	**0.58**	*	**0.99**
	**positive regulation of release of cytochrome c**	**0.76**	**	**	**	**	**	**	**
	**Response to ischemia**	*	**	**	**	**0.58**	**0.36**	**0.17**	**0.84**

Discriminant analysis performed on biological clusters related to oxidation-reduction, immune response and apoptosis after partial optic nerve injury. Table illustrating the p-values of multiple comparisons of genes from dorsal and ventral retina from normal (norm) uninjured animals and at days 1 or 7 following PT injury associated with A: oxidation reduction (65 genes) and glutathione metabolic process (6 genes). B: Innate immune response (70 genes): adaptive immune response (32 genes); cytokine signalling pathway (30 genes). C) Apoptosis (81 genes): extrinsic apoptotic pathway (16 genes); intrinsic apoptotic pathway (18 genes); intrinsic apoptotic signalling pathway in response to ER (6 genes); intrinsic apoptotic signalling in response to DNA damage (10 genes); regulation of mitochondrial membrane permeability (7 genes); positive regulation of release of cytochrome c (6 genes); response to ischemia (5 genes). Comparisons were made between the dorsal and ventral retinal tissue of normal uninjured animals (NormD) and (NormV) respectively, dorsal and ventral retina at day 1 following PT (D1PTD) and (D1PT V) respectively and dorsal and ventral retina at day 7 following PT (D7PTD) and (D7PTV) respectively. P-values <0.001 are depicted by **, which P-values <0.05 are depicted by *. Non-significant differences are in grey.

#### Immune response

Discriminant analysis of immune response variables demonstrated that following PT injury both dorsal and ventral retina were significantly different from normal control retina at days 1 and 7 post injury (Table 4B). In addition, there were significant differences between dorsal and ventral retina at days 1 and 7 after PT. The expression of genes associated with the innate immune response in areas vulnerable to secondary degeneration was significantly different from time matched dorsal retina. In contrast, changes in genes associated with the adaptive immune response were not significantly different between dorsal and ventral retina following PT at days 1 and 7. Thus, the recruited immune response appears to be similar in retinal tissue affected by PT injury and tissue vulnerable to secondary degeneration (Table 4B). We also analyzed immune response genes specifically associated with cytokine-mediated signalling pathways and similarly identified that following PT injury, retinal gene expression was significantly different from the expression of genes in injured retinal tissue from uninjured animals (Table 4B).

#### The expression of apoptotic genes in dorsal and ventral retina

Sub clusters of the apoptosis gene set were generated and the top biological processes subjected to discriminant analysis. We initially performed discriminant analysis on all the genes associated with apoptosis and all possible pair wise comparisons were significantly different (p ≤ 0.0001). Similarly, extrinsic and intrinsic apoptotic pathways, which were enriched by 16 and 17 genes respectively, were also significantly different between all groups (Table 4C). Genes in the intrinsic apoptotic pathway were further categorized into response to endoplasmic reticulum (ER) stress and response to DNA damage and these were enriched in 6 and 12 genes respectively. Discriminant analysis on the group of genes associated with response to ER stress indicated a significant difference between both dorsal and ventral retina in injured compared to respective regions in normal animals (p ≤ 0.0006). This difference was clear at day 1 following PT injury (p ≤ 0.0001) but not at day 7, although at this time expression remained different to control. Similarly, genes associated with intrinsic apoptotic signalling mediated by response to DNA damage were significantly different in dorsal and ventral retina following injury in comparison to uninjured normal animals at days 1 and 7 post PT. It is important to note that after PT, dorsal retina was significantly different from ventral retina at day 1 but not at day 7.

Under the umbrella of genes associated with the apoptotic pathway, 7 genes are closely associated with regulating mitochondrial permeability. Discriminant analysis indicated significant differences in the expression of these genes between PT injured and normal uninjured animals. Although a significant difference was observed between dorsal and ventral retina at day 1 following ON injury, by day 7 dorsal and ventral retina expression values were not different from each other (Table 4C).

Further analysis of genes within the apoptosis gene set revealed enrichment of 6 genes associated with positive regulation of release of cytochrome c from the mitochondrial membrane. Discriminant analysis showed no significant difference between injured and uninjured dorsal retina at day 1, and a significant difference at day 7. Interestingly we observed significant differences between ventral retina of injured and uninjured animals at both days 1 and 7 following injury (Table 4C). This suggests the release of cytochrome c from the mitochondria plays a more relevant role in driving apoptosis in secondary degeneration than following the primary injury. Finally, 5 differentially expressed genes associated with apoptosis were associated with response to ischemia, but there was no significant difference between dorsal and ventral tissue at either day 1 or 7 after PT.

#### Verification of microarray data

**Fig 4:**
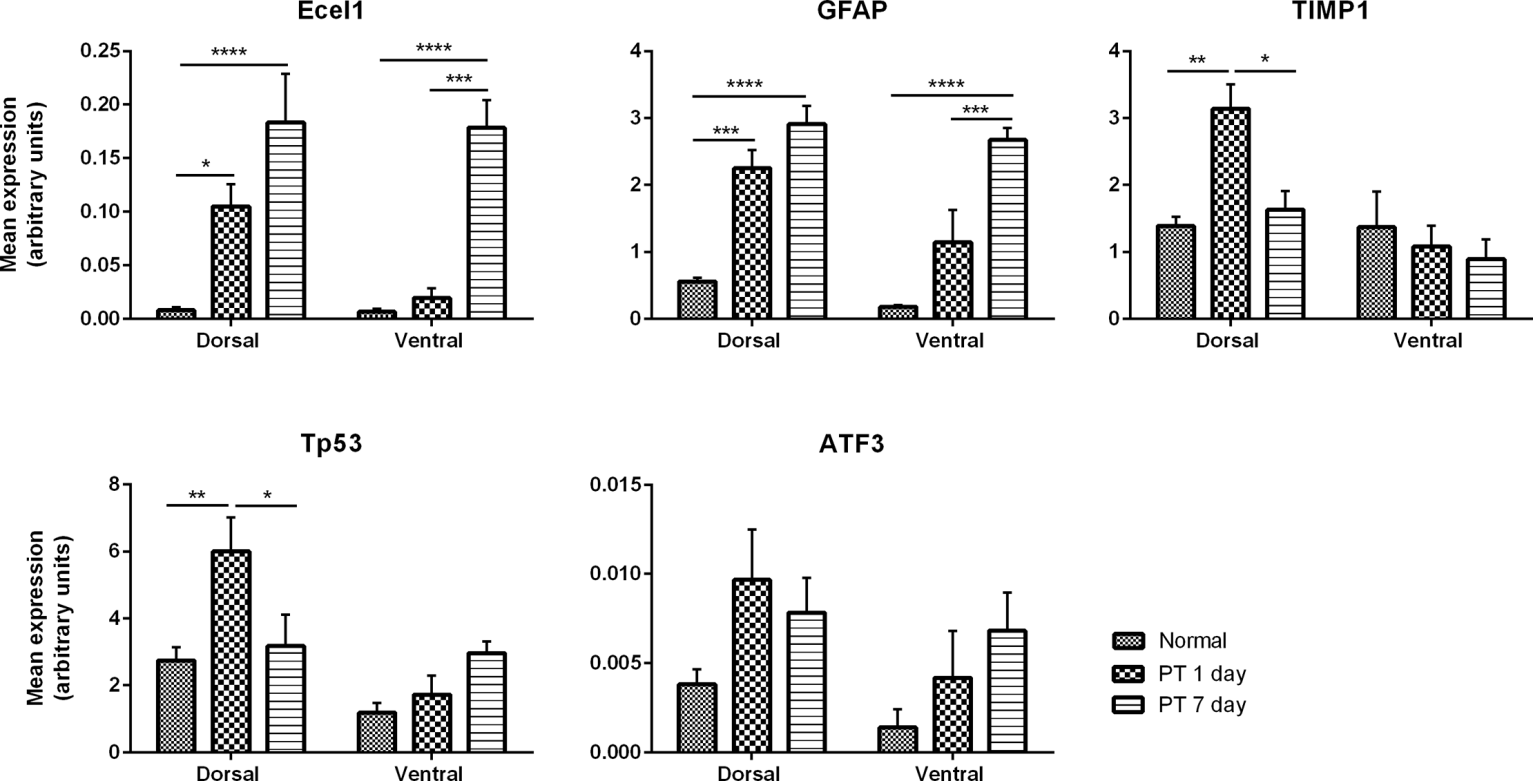
Real-time quantitative PCR confirmation of microarray findings. RT-qPCR analysis of 5 genes that were shown by microarray analysis to be differentially regulated following PT injury in dorsal and ventral retina at days 1 and 7. Data are expressed as mean±SEM expression of calculated concentration and normalised to 3 housekeeper genes. The same retinal RNA tissue was used as that for microarray analysis. (A) Endothelin converting enzyme-like 1 (Ecel1). (B) Glial fibrillary acidic protein (GFAP). (C) Tissue inhibitor of metalloproteinases 1 (Timp1). (D) Tumor protein 53 (Tp53). (E) Activating transcription factor 3 (ATF3). Statistical significance is indicated by *asterisks* (* p-value < 0.05, ** p-value < 0.01, *** p-value <0.001, **** p-value <0.0001).

Once genes were annotated and clustered, 5 genes with robust changes or genes related to apoptosis were chosen for array validation using qRT-PCR (Fig 4). The genes assessed were Ecel1, TIMP1, GFAP, ATF3 and Tp53. Three of the selected genes, namely Ecel1, TIMP1 and GFAP represented the robust gene changes as indicated in the microarray analysis; TIMP1 and ATF3 were also identified in several gene groups in GSEA, as was Tp53. Changes in genes after PT injury, assessed using qRT-PCR followed similar trends to the microarray data, however there were differences in net expression (Fig 4), likely due to the different experimental method and sensitivities. Furthermore, normalization protocols vary for each dataset. The array normalization protocol relies on mathematical algorithms, while qRT-PCR was normalized to the geometric mean of three housekeeper genes. Despite this, for the majority of genes studied using qRT-PCR, regulation was similar to the microarray experiment, validating the latter. Exceptions were, for example, the expression levels of GFAP, which remained unchanged at 1 day 1 PT and was upregulated at day 7 PT in ventral retina when analyzed by qRT-PCR, but was decreased and remained unchanged in the array analysis respectively. The increase may have been detected using qRT-PCR due to the filtration process in the pair-wise comparison; analysis of array data where comparison was conducted on normalized raw expression intensity of GFAP gene revealed significant increase when compared to uninjured normal retina (p ≤ 0.001).

#### Comparison of protein immunoreactivity with microarray mRNA data

**Fig 5:**
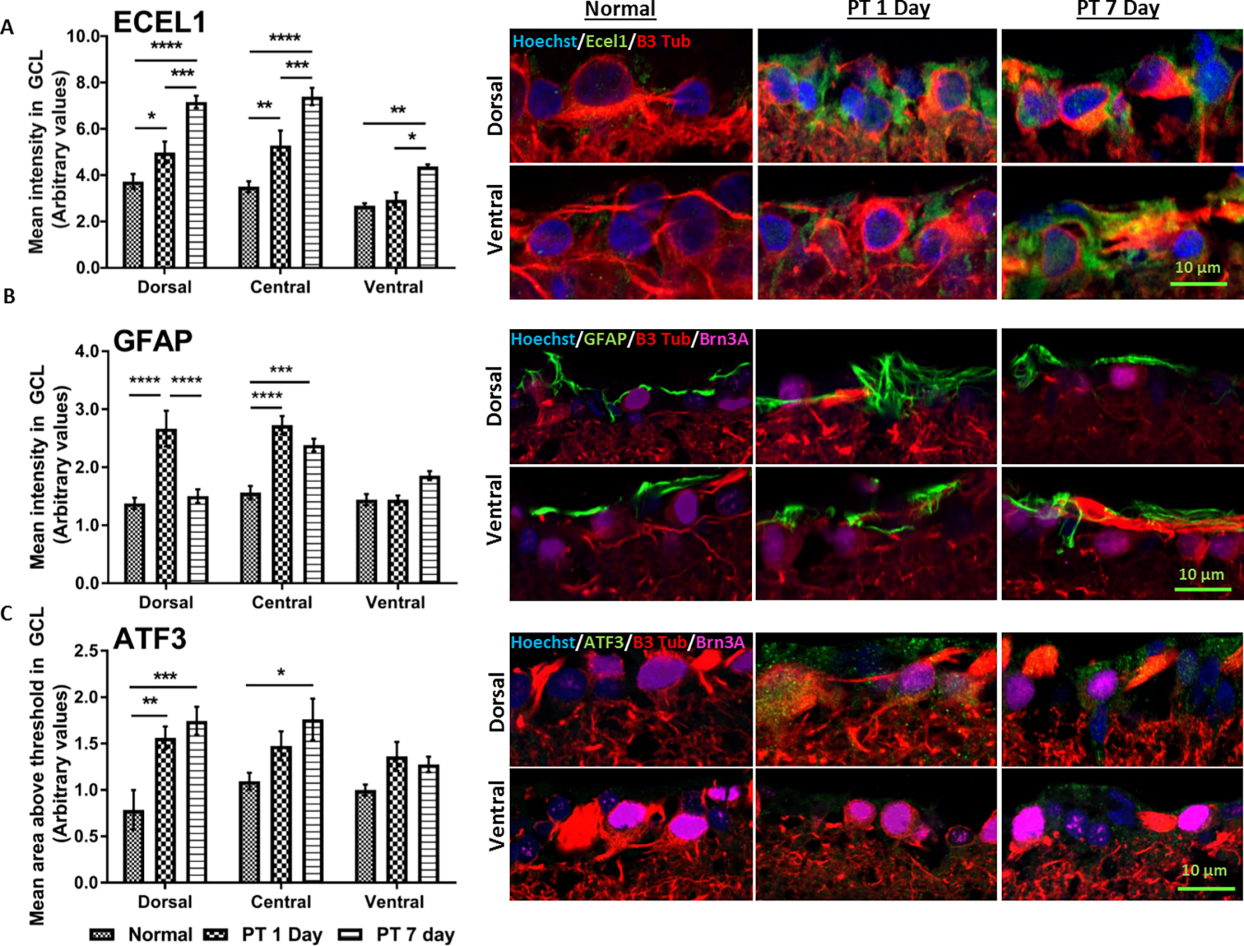
Immunoreactivity of Ecel 1 GFAP and ATF3 in retinal sections. **(**A) Endothelin converting enzyme-like 1 (Ecel1), (B) Glial fibrillary acidic protein (GFAP) and (C) Activating transcription factor-3 (ATF3); representative images shown for dorsal and ventral retina. Semi-quantification of immunointensity was performed on the retinal ganglion cell layer (GCL) in dorsal, central and ventral retina on normal uninjured animals and the retina of animals at 1 and 7 days following PT. Error bars show the standard error of the mean (SEM) for each experiment. Statistically analysis conducted on each region and significant differences between retinal tissue of injured and uninjured ON are indicated by *asterisks* (* p-value < 0.05, ** p-value < 0.01, *** p-value <0.001). Scale bar = 10 μm.

To assess the extent to which observed gene expression changes were reflected in protein changes following PT injury, we performed immunohistochemical analysis of Ecel1, GFAP and ATF3 on dorsal, central and ventral retinal sections of normal uninjured animals and at days 1 and 7 following PT. The use of immunohistochemistry on cross sections of the retina maintains topographic separation of tissue and identifies cell types including RGCs; semi-quantification was conducted on the ganglion cell layer. We found instances of both complementary as well as non-correlative changes. For example, Ecel1 immunoreactivity increased significantly in dorsal and central retina at days 1 and 7 after PT injury (p ≤ 0.05). In ventral retina, a significant (p ≤ 0.01) increase in Ecel1 protein immunolabeling was observed only at day 7, which matched a significant two-fold increase in mRNA levels of Ecel1 in ventral retina at day 7 following PT injury when assessed with PCR and when comparing normalised raw expression data (Fig 5A). In dorsal retina at day 1 after PT, there was a significant (p ≤ 0.01) increase in GFAP immunoreactivity as well as an increase in GFAP gene expression; however, although mRNA levels remained elevated compared to controls at day 7(qRT_PCR), GFAP protein immunolabeling returned to essentially normal levels. GFAP immunoreactivity was not changed in ventral retina at either time point after injury, although GFAP mRNA was significantly decreased at day 1 in the array analysis. The level of ATF3 immunoreactivity reflects array mRNA expression in dorsal retina after injury (Fig 5, Table 3). There was a significant increase in ATF3 protein immuno-intensity (p ≤ 0.01) in dorsal retina at day 1 and 7 and in central retina at day 7 post injury (p ≤ 0.05). Finally, ATF3 immunoreactivity was not different in ventral retina at days 1 and 7 after injury, despite significant increases in mRNA levels at day 1 (Table 3).

## Discussion

This study documents the spatial and temporal profile of the retinal transcriptome after partial transection (PT) injury to the adult rat optic nerve, a model for CNS injury. Such injuries trigger a plethora of gene expression changes in the dorsal retina affected by the primary injury that in many cases is different to the response in retinal tissue vulnerable to secondary degeneration. Although molecular changes following optic nerve transection or crush have been described [20, 31–33], this is the first report to provide a comprehensive survey of the expression changes in retinal tissue associated with primary injury versus secondary degeneration, and assessed at different times after the initial trauma. We show that events that lead to secondary degeneration are initially different to primary injury and result in the death of RGCs by mechanisms that are distinct, presumably reflecting different degenerative processes. These differences in molecular response to trauma are likely to account for the protracted period of RGC death consistently seen after PT of the optic nerve [6, 9, 22, 26].

We performed RNA extraction from retinal tissue at days 1 and 7 following PT of the optic nerve. An important aspect of the current study was the way retinal tissue was dissected out to enable different regions of the retina to be analysed separately. Pieces of retinal tissue either contained RGCs with axons directly transected after the primary PT injury, or contained RGCs with initially intact axons but located in regions vulnerable to secondary degeneration [7]. The advantage of using a microarray approach is that it does not require *a priori* assumptions about the genetic changes or molecular pathways involved. Further, microarray analysis using whole retinal tissue makes no assumptions as to which cells contribute to the degenerative events that follow ON injury, and the array characterizes the holistic genetic response to injury. Analysis of changes in purified RGC populations would be informative, although physical isolation of these neurons might itself subtly alter gene expression in these already injured neurons. Indeed, several studies indicate that the sequence of events leading to RGC death following ON injury may be strongly influenced by other cells residing in, or infiltrating, the retina [34, 35].

Here we describe the degenerative events of a heterogeneous cell population that is tissue specific rather than cell-specific in response to PT injury. The distribution and density of different cell types in the retina is likely to change after injury between day 1 and 7 in dorsal compared to ventral retina [9, 36]. Further, identified gene changes may reflect differential activation of intrinsic and infiltrating inflammatory and immune cells within the retinal tissue. Although a microarray analysis of mixed cellular population is undoubtedly ‘noisy’, it aids in identifying the gene expression changes that occur because of the initial trauma and that contribute to overall damage. In order to reduce the inherent noise associated with microarray data, we employed stringent statistical analyses and confirmatory studies. Firstly, three biological replicates were used, common in microarray studies [27, 28]. Secondly, differentially expressed genes were rigorously selected based on statistical significance (p ≤ 0.05) and appropriate fold change cut off. Finally, confirmatory RT-PCR studies were used to validate a selected group of genes.

Similar to post-injury effects in other parts of the CNS [37], damage to the optic nerve resulted in robust upregulation of Ecel1 (endothelin converting enzyme-like 1, also known as damage induced neuronal endopeptidase DINE). Ecel1 is a neuron specific membrane bound metalloprotease, which is thought to be activated by the interaction of ATF3, cJun and Stat3 via SP1, with the Ecel1 promotor present in damaged neurons [38]. Ecel1 enhances the expression and activity of antioxidant enzymes such as Cu/Zn-superoxide dismutase (SOD), Mn-SOD, and glutathione peroxidase under conditions of oxidative stress in damaged neurons [37]. Expression of Ecel1 is induced in response to spinal, optic, cortical and thalamic injuries, and is restricted to damaged neurons rather than surrounding glial cells [37]. It is thought that a chemical or mechanical injury to the axon precedes and may be required to induce Ecel1 upregulation in the somata [37]. Here we report a substantial and rapid upregulation of Ecel1 levels in dorsal retina containing RGCs with axons directly affected by the PT injury. However, initially we did not detect significant changes in the expression of Ecel1 gene and protein levels in ventral retina, consistent with the fact that ventral axons remain initially intact after PT of the optic nerve. While the array analysis did not show any changes in Ecel1 gene expression, PCR and immunohistochemistry did demonstrate a significant increase in gene and protein levels respectively. The early activation of ATF3 and previously reported upregulation of cJun in ventral retina following PT injury [6], factors that precede Ecel1 activation [38], provide evidence that Ecel1 is likely to be associated with secondary degeneration and potentially enhance antioxidant activity of RGCs vulnerable to secondary degeneration.

The observed injury-response gene expression changes can essentially be separated into those uniquely expressed in dorsal retina, and therefore regulated by the primary injury, and those common to both dorsal and ventral retina, as well as a direct comparison between dorsal and central retina at days 1 and 7 following ON injury. We did not observe any genes differentially expressed only in ventral retina. In dorsal retina at day 1 and 7 following injury, the expression of crystallin genes was significantly downregulated when compared to uninjured neural tissue. There was a trend for crystallin family gene expression to be less downregulated in ventral compared to dorsal retina after injury, this difference was not statistically significant. Studies suggest crystallins, namely beta and gamma, play a role in regeneration following optic nerve injury and are thought to be involved in cell protection by enhancing cellular resistance to stress-induced apoptosis [39]. Following injury, several studies report dramatic upregulation of some members of the crystallin family, including following ischemia-reperfusion injury [40], light injury [41], mechanical injury [42]and optic nerve transection [32, 33]. Contrary to these observations, retinal gene expression profiles demonstrated a downregulation in the expression of crystallin members following optic nerve transection [43], in an animal model of glaucoma [44], and following experimental elevation of intraocular pressure [31]. It has been further demonstrated, through co-localization of crystallin expression and flurogold retrogradely labelled cells, that RGCs are the main cells expressing crystallins in the ganglion cell layer [45]. We also found a significant and dramatic downregulation of *Cryba1* at day 1, and *Cryba2*, and *Crygs* at day 7 in dorsal retinal tissue. The downregulation cannot be simply attributed to RGC death as our group and others demonstrate RGC loss begins at days 3–7 in retinal tissue whose axons are transected [22, 26]. Therefore, we argue the injury signal induced following PT injury may negatively regulate the expression of crystallin genes in the retina, in both neural tissue affected directly by ON injury and proximate tissue vulnerable to secondary degeneration, further reducing stress resistance capacity and facilitate degeneration.

One of the most enriched GSEA functional groups following PT injury was associated with the oxidation-reduction process. This was perhaps not surprising, as increased reactive species, anti-oxidant activity and oxidative damage in dorsal optic nerve is known to spread to areas of the nerve vulnerable to secondary degeneration following PT [6, 16, 22]. An increase in MnSOD immunoreactivity was also seen in central and ventral retina following PT injury associated with RGCs [6, 26]. We demonstrate increased levels of oxidative stress in retinal tissue impacted by the primary injury and early phases of secondary degeneration, suggesting that the early propagation of injury signals spread locally from injured RGCs, as well as vulnerable axons from the injury site. Interestingly, the lack of difference in expression profiles of genes associated with oxidative stress in ventral retina at day 7 relative to normal tissue suggests that tissue vulnerable to secondary degeneration remote from the injury may restore antioxidant and homeostatic mechanisms, despite ongoing oxidative damage at the injury site [15, 46]. This is exemplified by changes in the glutathione anti-oxidant metabolic process, which is different to uninjured tissue, in ventral retina vulnerable to secondary degeneration, at both days 1 and 7 post injury.

Interpreting the role of the immune response following CNS injury is complex, with the literature presenting a disparity between the inflammation that disrupts the maintenance of the CNS on the one hand, versus the inflammation that under appropriate conditions facilitates CNS repair. The inflammatory response is also suggested to play a critical role in the progression of secondary degeneration [4]. In this study, we provide evidence for significant changes in the expression profiles of genes associated with the adaptive and innate immune response, as well as cytokine mediated activity in primary and secondary degeneration. Regulation of genes associated with the adaptive immune response seems to be temporally similar in primary and secondary degeneration. Conversely, activation of the innate immune system is different in dorsal compared to ventral retina, consistent with observed temporal differences in innate inflammatory cell recruitment and infiltration to dorsal retina [6]

Different rates of RGC death in dorsal compared to ventral retina are a hallmark of primary and secondary degeneration following PT injury [22, 26]. Our new data are consistent with previous observations demonstrating significant RGC death in the dorsal retina as early as day 3 following PT, spreading to ventral retina by 3 weeks [22, 26]. Secondary RGC death following PT injury is characterized by multiple mechanisms of death, with predominantly necrotic morphologies as well as caspase-3 mediated apoptosis [26]. Elevated levels of pro-apoptotic genes Bax and BAD, and downregulation of anti-apoptotic gene Bcl-2 in primary and secondary degeneration are also observed [22]. The present study supports these findings, with differential expression and enrichment of apoptosis associated genes in the mixed cellular environment of retina as early as day 1 following PT in dorsal retina and delayed to day 7 in ventral retina. Direct comparison between differentially expressed genes in dorsal and ventral retina following ON injury lends further support for apoptosis associated genes being enriched early after injury. Using GSEA and PANTHER we also demonstrate that retinal tissue in dorsal and ventral regions is vulnerable to both intrinsic and extrinsic apoptotic mechanisms following PT. However, it is important to note that gene sets associated with intrinsic and extrinsic apoptotic pathways show significantly different expression patterns between dorsal and ventral retina at days 1 and 7. Similarly, the gene expression pattern of apoptosis associated genes is significantly different within dorsal and ventral retina across time. This not only suggests differing apoptosis mechanisms between primary and secondary degeneration, but also within retinal regions across time, at least within the first week of injury, and before death of RGCs in ventral retina reaches significant levels.

The expression profile of death-associated genes, including those relating to ER stress, DNA damage and regulation of mitochondrial membrane permeability, were significantly different between injured and uninjured tissue, as well as between dorsal and ventral retina at day 1 post PT. Interestingly, we did not observe differences in the expression of death-associated genes between dorsal and ventral retinal tissue at day 7 post injury, suggesting ventral retinal tissue begins to behave transcriptionally, and potentially mechanistically, similar to dorsal retina. In particular, we observed a difference between the expression of genes that are associated with positive release of cytochrome *c* in ventral retina following PT at day 1 compared to retina of uninjured animals, suggesting that the cytochrome *c* mediated apoptotic pathway is specifically associated with secondary degeneration. Conversely, response to ischemia appears to be a global injury response independent of degenerative mechanism.

Understanding the propagating genetic changes and molecular pathways following axonal lesion is key to the characterization of pharmacological intervention. Optic nerve injury neatly represents *in-vivo* the events associated with neuronal apoptosis in the CNS and often the conclusions drawn from axotomized RGC apoptotic death can be generalised for neuronal death following brain trauma [47]. In conclusion, the data confirm that a CNS tissue directly affected by an injury experiences a more acute and pronounced injury response than tissue vulnerable to secondary degeneration. Nonetheless, in retina both primary and secondary injury resulted in the upregulation of genes linked to cell death, but differences in the nature of these gene changes suggest that death occurred *via* different mechanisms. Presumably the delayed changes in regulatory genes associated with cell death in ventral retina accounted for the delayed and slower degeneration of RGCs [99]. Further assessments at a later time point, confined to the ganglion cell layer, would allow more direct comparison of RGC death associated genes in primary and secondary degeneration. Taken together with previous findings, we demonstrate, following CNS injury, responses to primary and secondary degenerative events are genetically and mechanistically different, although in the retina at least, RGC viability is eventually compromised throughout. The complex molecular events that trigger secondary degeneration after CNS injury highlight the limited and narrowing time window for therapeutic intervention.

## Supporting information

S1 Text **A:** GSEA output on enriched functional groups comparing retina from dorsal day 1 PT to uninjured dorsal retina.
**B:** GSEA output on enriched functional groups comparing retina from dorsal day 7 PT to uninjured dorsal retina.
**C:** GSEA output on enriched functional groups comparing retina from ventral day 1 PT to uninjured ventral retina.
**D:** GSEA output on enriched functional groups comparing retina from ventral day 7 PT to uninjured ventral retina.
(DOCX)

## Abbreviations

CNS: central nervous system
PT: Partial transection
PVG: Piebald Viral Glaxo
ON: optic nerve
RGC: retinal ganglion cell
IAP-1: inhibitor of apoptosis protein-1
GADD45α: Growth arrest and DNA damage inducible protein 45α
CDK2: cyclin-dependent kinase 2
ei24: etoposide-induced protein 2.4 homolog
RMA: Robust Multichip Average
GSEA: Gene set enrichment analysis
GO: Gene Ontology
OCT: optimal cutting temperature
ECEL1: endothelin converting enzyme-like 1
TIMP1: metallopeptidase inhibitor 1
MT2A: metallothionein 2A
CD74: major histocompatibility complex, class II invariant chain
GFAP: glial fibrillary acidic protein
RGR: retinal g-protein coupled receptor
TMEM47: transmembrane protein 47
ER: endoplasmic reticulum
DINE: damage induced neuronal endopeptidase
SOD: superoxide dismutase

## References

[1] Stoica BA, Faden AI. Cell death mechanisms and modulation in traumatic brain injury. Neurotherapeutics. 2010;7(1):3–12. doi: 10.1016/j.nurt.2009.10.023 ; PubMed Central PMCID: PMC2841970. 20129492 10.1016/j.nurt.2009.10.023 PMC2841970

[2] Oyinbo CA. Secondary injury mechanisms in traumatic spinal cord injury: a nugget of this multiply cascade. Acta Neurobiologiae Experimentalis. 2011;71(2):281–99. PubMed PMID: WOS:000293206400010. 21731081 10.55782/ane-2011-1848

[3] Liang ZJ, Zeng JS, Liu SR, Ling XY, Xu AD, Yu J, et al A prospective study of secondary degeneration following subcortical infarction using diffusion tensor imaging. Journal of Neurology Neurosurgery and Psychiatry. 2007;78(6):581–6. doi: 10.1136/jnnp.2006.099077 PubMed PMID: WOS:000246593900010. 17237143 10.1136/jnnp.2006.099077 PMC2077972

[4] Yoles E, Schwartz M. Degeneration of spared axons following partial white matter lesion: Implications for optic nerve neuropathies. Experimental Neurology. 1998;153(1):1–7. doi: 10.1006/exnr.1998.6811 PubMed PMID: WOS:000076181200001. 9743562 10.1006/exnr.1998.6811

[5] Levkovitch-Verbin H, Quigley HA, Martin KR, Zack DJ, Pease ME, Valenta DF. A model to study differences between primary and secondary degeneration of retinal ganglion cells in rats by partial optic nerve transection. Invest Ophthalmol Vis Sci. 2003;44(8):3388–93. . 12882786 10.1167/iovs.02-0646

[6] Fitzgerald M, Bartlett CA, Harvey AR, Dunlop SA. Early events of secondary degeneration after partial optic nerve transection: an immunohistochemical study. J Neurotrauma. 2010;27(2):439–52. doi: 10.1089/neu.2009.1112 . 19852581 10.1089/neu.2009.1112

[7] Chan KC, Zhou IY, Liu SS, van der Merwe Y, Fan SJ, Hung VK, et al Longitudinal Assessments of Normal and Perilesional Tissues in Focal Brain Ischemia and Partial Optic Nerve Injury with Manganese-enhanced MRI. Sci Rep-Uk. 2017;7. doi: ARTN 43124 doi: 10.1038/srep43124 PubMed PMID: WOS:000394748100001. 28230106 10.1038/srep43124 PMC5322351

[8] Li HY, Liang YX, Chiu K, Yuan QJ, Lin B, Chang RCC, et al Lycium Barbarum (Wolfberry) Reduces Secondary Degeneration and Oxidative Stress, and Inhibits JNK Pathway in Retina after Partial Optic Nerve Transection. Plos One. 2013;8(7). doi: ARTN e68881 doi: 10.1371/journal.pone.0068881 PubMed PMID: WOS:000322391400029. 23894366 10.1371/journal.pone.0068881 PMC3716882

[9] Fitzgerald M, Payne SC, Bartlett CA, Evill L, Harvey AR, Dunlop SA. Secondary retinal ganglion cell death and the neuroprotective effects of the calcium channel blocker lomerizine. Invest Ophthalmol Vis Sci. 2009;50(11):5456–62. doi: 10.1167/iovs.09-3717 . 19474405 10.1167/iovs.09-3717

[10] Choi DW. Ionic dependence of glutamate neurotoxicity. J Neurosci. 1987;7(2):369–79. . 2880938 10.1523/JNEUROSCI.07-02-00369.1987 PMC6568907

[11] Katayama Y, Becker DP, Tamura T, Hovda DA. Massive increases in extracellular potassium and the indiscriminate release of glutamate following concussive brain injury. J Neurosurg. 1990;73(6):889–900. doi: 10.3171/jns.1990.73.6.0889 . 1977896 10.3171/jns.1990.73.6.0889

[12] Seifert G, Schilling K, Steinhauser C. Astrocyte dysfunction in neurological disorders: a molecular perspective. Nat Rev Neurosci. 2006;7(3):194–206. doi: 10.1038/nrn1870 . 16495941 10.1038/nrn1870

[13] Martin LJ. The mitochondrial permeability transition pore: a molecular target for amyotrophic lateral sclerosis therapy. Biochim Biophys Acta. 2010;1802(1):186–97. doi: 10.1016/j.bbadis.2009.07.009 ; PubMed Central PMCID: PMC2790555. 19651206 10.1016/j.bbadis.2009.07.009 PMC2790555

[14] Bernardi P, Krauskopf A, Basso E, Petronilli V, Blachly-Dyson E, Di Lisa F, et al The mitochondrial permeability transition from in vitro artifact to disease target. FEBS J. 2006;273(10):2077–99. doi: 10.1111/j.1742-4658.2006.05213.x . 16649987 10.1111/j.1742-4658.2006.05213.x

[15] Szymanski CR, Chiha W, Morellini N, Cummins N, Bartlett CA, O'Hare Doig RL, et al Paranode Abnormalities and Oxidative Stress in Optic Nerve Vulnerable to Secondary Degeneration: Modulation by 670 nm Light Treatment. Plos One. 2013;8(6):e66448 doi: 10.1371/journal.pone.0066448 ; PubMed Central PMCID: PMC3686728. 23840470 10.1371/journal.pone.0066448 PMC3686728

[16] O'Hare Doig RL, Bartlett CA, Maghzal GJ, Lam M, Archer M, Stocker R, et al Reactive species and oxidative stress in optic nerve vulnerable to secondary degeneration. Exp Neurol. 2014;261:136–46. doi: 10.1016/j.expneurol.2014.06.007 . 24931225 10.1016/j.expneurol.2014.06.007

[17] Moreno MC, Campanelli J, Sande P, Sanez DA, Keller Sarmiento MI, Rosenstein RE. Retinal oxidative stress induced by high intraocular pressure. Free Radic Biol Med. 2004;37(6):803–12. . 15384194 10.1016/j.freeradbiomed.2004.06.001

[18] Szydlowska K, Tymianski M. Calcium, ischemia and excitotoxicity. Cell Calcium. 2010;47(2):122–9. doi: 10.1016/j.ceca.2010.01.003 . 20167368 10.1016/j.ceca.2010.01.003

[19] Wells J, Kilburn MR, Shaw JA, Bartlett CA, Harvey AR, Dunlop SA, et al Early in vivo changes in calcium ions, oxidative stress markers, and ion channel immunoreactivity following partial injury to the optic nerve. J Neurosci Res. 2012;90(3):606–18. doi: 10.1002/jnr.22784 . 22038561 10.1002/jnr.22784

[20] Agudo M, Perez-Marin MC, Lonngren U, Sobrado P, Conesa A, Canovas I, et al Time course profiling of the retinal transcriptome after optic nerve transection and optic nerve crush. Mol Vis. 2008;14:1050–63. ; PubMed Central PMCID: PMC2426719. 18552980 PMC2426719

[21] Agudo M, Perez-Marin MC, Sobrado-Calvo P, Lonngren U, Salinas-Navarro M, Canovas I, et al Immediate upregulation of proteins belonging to different branches of the apoptotic cascade in the retina after optic nerve transection and optic nerve crush. Invest Ophthalmol Vis Sci. 2009;50(1):424–31. doi: 10.1167/iovs.08-2404 . 18775855 10.1167/iovs.08-2404

[22] Levkovitch-Verbin H, Dardik R, Vander S, Melamed S. Mechanism of retinal ganglion cells death in secondary degeneration of the optic nerve. Exp Eye Res. 2010;91(2):127–34. doi: 10.1016/j.exer.2009.11.014 . 19951705 10.1016/j.exer.2009.11.014

[23] Levkovitch-Verbin H, Spierer O, Vander S, Dardik R. Similarities and differences between primary and secondary degeneration of the optic nerve and the effect of minocycline. Graefes Archive for Clinical and Experimental Ophthalmology. 2011;249(6):849–57. doi: 10.1007/s00417-010-1608-2 PubMed PMID: WOS:000291166600009. 21229256 10.1007/s00417-010-1608-2

[24] Bodeutsch N, Siebert H, Dermon C, Thanos S. Unilateral injury to the adult rat optic nerve causes multiple cellular responses in the contralateral site. J Neurobiol. 1999;38(1):116–28. . 10027567 10.1002/(sici)1097-4695(199901)38:1<116::aid-neu9>3.0.co;2-f

[25] Sharma A, Pollett MA, Plant GW, Harvey AR. Changes in mRNA Expression of Class 3 Semaphorins and Their Receptors in the Adult Rat Retino-Collicular System after Unilateral Optic Nerve Injury. Investigative Ophthalmology & Visual Science. 2012;53(13):8367–77. doi: 10.1167/iovs.12-10799 PubMed PMID: WOS:000313056000060. 23139269 10.1167/iovs.12-10799

[26] Fitzgerald M, Bartlett CA, Evill L, Rodger J, Harvey AR, Dunlop SA. Secondary degeneration of the optic nerve following partial transection: the benefits of lomerizine. Exp Neurol. 2009;216(1):219–30. doi: 10.1016/j.expneurol.2008.11.026 . 19118550 10.1016/j.expneurol.2008.11.026

[27] Liedtke T, Naskar R, Eisenacher M, Thanos S. Transformation of adult retina from the regenerative to the axonogenesis state activates specific genes in various subsets of neurons and glial cells. Glia. 2007;55(2):189–201. doi: 10.1002/glia.20447 . 17078023 10.1002/glia.20447

[28] Panagis L, Zhao X, Ge Y, Ren L, Mittag TW, Danias J. Retinal gene expression changes related to IOP exposure and axonal loss in DBA/2J mice. Investigative ophthalmology & visual science. 2011;52(11):7807–16. doi: 10.1167/iovs.10-7063 ; PubMed Central PMCID: PMC3335131. 21908583 10.1167/iovs.10-7063 PMC3335131

[29] Ritchie ME, Phipson B, Wu D, Hu Y, Law CW, Shi W, et al limma powers differential expression analyses for RNA-sequencing and microarray studies. Nucleic Acids Res. 2015;43(7):e47 doi: 10.1093/nar/gkv007 ; PubMed Central PMCID: PMC4402510. 25605792 10.1093/nar/gkv007 PMC4402510

[30] Mi H, Muruganujan A, Casagrande JT, Thomas PD. Large-scale gene function analysis with the PANTHER classification system. Nat Protoc. 2013;8(8):1551–66. doi: 10.1038/nprot.2013.092 . 23868073 10.1038/nprot.2013.092 PMC6519453

[31] Ahmed F, Brown KM, Stephan DA, Morrison JC, Johnson EC, Tomarev SI. Microarray analysis of changes in mRNA levels in the rat retina after experimental elevation of intraocular pressure. Invest Ophthalmol Vis Sci. 2004;45(4):1247–58. . 15037594 10.1167/iovs.03-1123

[32] Piri N, Kwong JM, Song M, Elashoff D, Caprioli J. Gene expression changes in the retina following optic nerve transection. Mol Vis. 2006;12:1660–73. . 17200666

[33] Yang Z, Quigley HA, Pease ME, Yang Y, Qian J, Valenta D, et al Changes in gene expression in experimental glaucoma and optic nerve transection: the equilibrium between protective and detrimental mechanisms. Invest Ophthalmol Vis Sci. 2007;48(12):5539–48. doi: 10.1167/iovs.07-0542 . 18055803 10.1167/iovs.07-0542

[34] Bosco A, Inman DM, Steele MR, Wu G, Soto I, Marsh-Armstrong N, et al Reduced retina microglial activation and improved optic nerve integrity with minocycline treatment in the DBA/2J mouse model of glaucoma. Invest Ophthalmol Vis Sci. 2008;49(4):1437–46. doi: 10.1167/iovs.07-1337 . 18385061 10.1167/iovs.07-1337

[35] Nakazawa T, Takeda M, Lewis GP, Cho KS, Jiao J, Wilhelmsson U, et al Attenuated glial reactions and photoreceptor degeneration after retinal detachment in mice deficient in glial fibrillary acidic protein and vimentin. Invest Ophthalmol Vis Sci. 2007;48(6):2760–8. doi: 10.1167/iovs.06-1398 ; PubMed Central PMCID: PMCPMC2613948. 17525210 10.1167/iovs.06-1398 PMC2613948

[36] Levkovitch-Verbin H, Quigley HA, Martin KRG, Zack DJ, Pease ME, Valenta DF. A model to study differences between primary and secondary degeneration of retinal ganglion cells in rats by partial optic nerve transection. Investigative Ophthalmology & Visual Science. 2003;44(8):3388–93. doi: 10.1167/iovs.02-0646 PubMed PMID: WOS:000184383500020. 12882786 10.1167/iovs.02-0646

[37] Kiryu-Seo S, Sasaki M, Yokohama H, Nakagomi S, Hirayama T, Aoki S, et al Damage-induced neuronal endopeptidase (DINE) is a unique metallopeptidase expressed in response to neuronal damage and activates superoxide scavengers. Proc Natl Acad Sci U S A. 2000;97(8):4345–50. doi: 10.1073/pnas.070509897 ; PubMed Central PMCID: PMC18244. 10759559 10.1073/pnas.070509897 PMC18244

[38] Kiryu-Seo S, Kato R, Ogawa T, Nakagomi S, Nagata K, Kiyama H. Neuronal injury-inducible gene is synergistically regulated by ATF3, c-Jun, and STAT3 through the interaction with Sp1 in damaged neurons. J Biol Chem. 2008;283(11):6988–96. doi: 10.1074/jbc.M707514200 . 18192274 10.1074/jbc.M707514200

[39] Piri N, Song M, Kwong JM, Caprioli J. Modulation of alpha and beta crystallin expression in rat retinas with ocular hypertension-induced ganglion cell degeneration. Brain Res. 2007;1141:1–9. doi: 10.1016/j.brainres.2006.11.095 . 17316577 10.1016/j.brainres.2006.11.095

[40] Yoshimura N, Kikuchi T, Kuroiwa S, Gaun S. Differential temporal and spatial expression of immediate early genes in retinal neurons after ischemia-reperfusion injury. Invest Ophthalmol Vis Sci. 2003;44(5):2211–20. . 12714663 10.1167/iovs.02-0704

[41] Sakaguchi H, Miyagi M, Darrow RM, Crabb JS, Hollyfield JG, Organisciak DT, et al Intense light exposure changes the crystallin content in retina. Exp Eye Res. 2003;76(1):131–3. . 12589783 10.1016/s0014-4835(02)00249-x

[42] Vazquez-Chona F, Song BK, Geisert EE Jr. Temporal changes in gene expression after injury in the rat retina. Invest Ophthalmol Vis Sci. 2004;45(8):2737–46. doi: 10.1167/iovs.03-1047 ; PubMed Central PMCID: PMC2821791. 15277499 10.1167/iovs.03-1047 PMC2821791

[43] Munemasa Y, Kwong JM, Caprioli J, Piri N. The role of alphaA- and alphaB-crystallins in the survival of retinal ganglion cells after optic nerve axotomy. Invest Ophthalmol Vis Sci. 2009;50(8):3869–75. doi: 10.1167/iovs.08-3138 . 19279307 10.1167/iovs.08-3138

[44] Steele MR, Inman DM, Calkins DJ, Horner PJ, Vetter ML. Microarray analysis of retinal gene expression in the DBA/2J model of glaucoma. Invest Ophthalmol Vis Sci. 2006;47(3):977–85. doi: 10.1167/iovs.05-0865 . 16505032 10.1167/iovs.05-0865

[45] Piri N, Song M, Kwong JMK, Caprioli J. Modulation of alpha and beta crystallin expression in rat retinas with ocular hypertension-induced ganglion cell degeneration. Brain Research. 2007;1141:1–9. doi: 10.1016/j.brainres.2006.11.095 PubMed PMID: WOS:000245491700001. 17316577 10.1016/j.brainres.2006.11.095

[46] Bidmon HJ, Kato K, Schleicher A, Witte OW, Zilles K. Transient increase of manganese-superoxide dismutase in remote brain areas after focal photothrombotic cortical lesion. Stroke. 1998;29(1):203–10; discussion 11. . 9445352 10.1161/01.str.29.1.203

[47] Weishaupt JH, Bahr M. Degeneration of axotomized retinal ganglion cells as a model for neuronal apoptosis in the central nervous system—molecular death and survival pathways. Restor Neurol Neurosci. 2001;19(1–2):19–27. . 12082226

