# Supplementary material for: Retinal genes are differentially expressed in areas of primary versus secondary degeneration following partial optic nerve injury
Source: PLoS One. 2018 Feb 9;13(2):e0192348. doi: 10.1371/journal.pone.0192348 (PMC5806857; doi:10.1371/journal.pone.0192348)
Supplement: S1 Text — A: GSEA output on enriched functional groups comparing retina from dorsal day 1 PT to uninjured dorsal retina. B: GSEA output on enriched functional groups comparing retina from dorsal day 7 PT to uninjured dorsal retina. C: GSEA output on enriched functional groups comparing retina from ventral day 1 PT to uninjured ventral retina. D: GSEA output on enriched functional groups comparing retina from ventral day 7 PT to uninjured ventral retina. (DOCX) [file pone.0192348.s001.docx]

***S1A Table: GSEA output on enriched functional groups comparing retina from dorsal day 1 PT to uninjured dorsal retina.***

| **Name** | **# of Entities** | **p-value** | **Hit type** |
| --- | --- | --- | --- |
| **oxidation-reduction process** | 90 | 0.0404 | biological_process |
| **apoptotic process** | 83 | 0.0009 | biological_process |
| **immune response** | 70 | 0.0355 | biological_process |
| **cell surface** | 62 | 0.0317 | cellular_component |
| **cell adhesion** | 57 | 0.0220 | biological_process |
| **negative regulation of cell proliferation** | 56 | 0.0087 | biological_process |
| **blood coagulation** | 56 | 0.0474 | biological_process |
| **metabolic process** | 52 | 0.0285 | biological_process |
| **response to organic cyclic compound** | 48 | 0.0376 | biological_process |
| **immune response** | 43 | 0.0187 | biological_process |
| **miRNAs** | 40 | 0.0006 | Pathway Studio Ontology |
| **extracellular matrix** | 39 | 0.0004 | cellular_component |
| **positive regulation of apoptotic process** | 39 | 0.0224 | biological_process |
| **immune system process** | 38 | 0.0143 | biological_process |
| **response to hypoxia** | 36 | 0.0497 | biological_process |
| **proteinaceous extracellular matrix** | 35 | 0.0003 | cellular_component |
| **cytokine-mediated signaling pathway** | 33 | 0.0144 | biological_process |
| **regulation of apoptotic process** | 27 | 0.0034 | biological_process |
| **response to organic substance** | 26 | 0.0290 | biological_process |
| **cell death** | 25 | 0.0113 | biological_process |
| **RNA metabolic process** | 25 | 0.0406 | biological_process |
| **positive regulation of angiogenesis** | 23 | 0.0014 | biological_process |
| **response to stress** | 22 | 0.0190 | biological_process |
| **ubiquitin protein ligase binding** | 19 | 0.0391 | molecular_function |
| **electron carrier activity** | 18 | 0.0052 | molecular_function |
| **hemopoiesis** | 17 | 0.0100 | biological_process |
| **response to cAMP** | 17 | 0.0255 | biological_process |
| **sarcolemma** | 16 | 0.0201 | cellular_component |
| **positive regulation of peptidyl-tyrosine phosphorylation** | 15 | 0.0077 | biological_process |
| **cellular response to growth factor stimulus** | 15 | 0.0205 | biological_process |
| **antigen processing and presentation of exogenous peptide antigen via MHC class I** | 15 | 0.0333 | biological_process |
| **neuron differentiation** | 14 | 0.0130 | biological_process |
| **leukocyte migration** | 14 | 0.0218 | biological_process |
| **Z disc** | 14 | 0.0271 | cellular_component |
| **protease binding** | 14 | 0.0375 | molecular_function |
| **G1-S transition of mitotic cell cycle** | 14 | 0.0382 | biological_process |
| **iron ion binding** | 14 | 0.0432 | molecular_function |
| **lysosomal lumen** | 13 | 0.0001 | cellular_component |
| **heme binding** | 13 | 0.0071 | molecular_function |
| **regulation of immune response** | 13 | 0.0223 | biological_process |
| **cartilage development** | 13 | 0.0247 | biological_process |
| **vitamin metabolic process** | 13 | 0.0466 | biological_process |
| **kidney development** | 12 | 0.0087 | biological_process |
| **defense response to virus** | 12 | 0.0099 | biological_process |
| **Aromatic amino acid metabolism** | 12 | 0.0223 | Pathway Studio Ontology |
| **endomembrane system** | 12 | 0.0257 | cellular_component |
| **response to retinoic acid** | 12 | 0.0478 | biological_process |
| **protein heterooligomerization** | 11 | 0.0234 | biological_process |
| **protein dephosphorylation** | 11 | 0.0256 | biological_process |
| **cellular response to hormone stimulus** | 11 | 0.0465 | biological_process |
| **cellular response to hypoxia** | 10 | 0.0103 | biological_process |
| **response to nicotine** | 10 | 0.0300 | biological_process |
| **basement membrane** | 10 | 0.0451 | cellular_component |
| **DNA damage response, signal transduction by p53 class mediator resulting in cell cycle arrest** | 9 | 0.0209 | biological_process |
| **negative regulation of NF-kappaB transcription factor activity** | 9 | 0.0340 | biological_process |
| **male gonad development** | 9 | 0.0345 | biological_process |
| **positive regulation of neuron apoptotic process** | 9 | 0.0345 | biological_process |
| **protein polyubiquitination** | 8 | 0.0048 | biological_process |
| **Extracellular matrix polymerization** | 8 | 0.0116 | Pathway Studio Ontology |
| **blood vessel remodeling** | 8 | 0.0223 | biological_process |
| **chaperone binding** | 8 | 0.0298 | molecular_function |
| **cell junction assembly** | 8 | 0.0308 | biological_process |
| **cellular response to interleukin-1** | 8 | 0.0372 | biological_process |
| **extracellular matrix structural constituent** | 7 | 0.0006 | molecular_function |
| **positive regulation of extrinsic apoptotic signaling pathway** | 7 | 0.0029 | biological_process |
| **complement activation, classical pathway** | 7 | 0.0186 | biological_process |
| **regulation of ubiquitin-protein ligase activity involved in mitotic cell cycle** | 7 | 0.0322 | biological_process |
| **negative regulation of ubiquitin-protein ligase activity involved in mitotic cell cycle** | 7 | 0.0322 | biological_process |
| **positive regulation of ubiquitin-protein ligase activity involved in mitotic cell cycle** | 7 | 0.0322 | biological_process |
| **anaphase-promoting complex-dependent proteasomal ubiquitin-dependent protein catabolic process** | 7 | 0.0322 | biological_process |
| **beta-catenin binding** | 7 | 0.0391 | molecular_function |
| **negative regulation of neuron projection development** | 7 | 0.0406 | biological_process |
| **response to electrical stimulus** | 7 | 0.0443 | biological_process |
| **cellular response to drug** | 6 | 0.0023 | biological_process |
| **T cell differentiation in thymus** | 6 | 0.0109 | biological_process |
| **positive regulation of intrinsic apoptotic signaling pathway** | 6 | 0.0152 | biological_process |
| **positive regulation of cell death** | 6 | 0.0162 | biological_process |
| **RNA polymerase II core promoter sequence-specific DNA binding** | 6 | 0.0240 | molecular_function |
| **regulation of cellular amino acid metabolic process** | 6 | 0.0303 | biological_process |
| **peptidyl-serine phosphorylation** | 6 | 0.0359 | biological_process |
| **cytokine receptor activity** | 5 | 0.0034 | molecular_function |
| **negative regulation of fibroblast proliferation** | 5 | 0.0238 | biological_process |
| **cellular response to glucose starvation** | 5 | 0.0268 | biological_process |
| **antigen processing and presentation** | 5 | 0.0293 | biological_process |
| **potassium channel regulator activity** | 5 | 0.0407 | molecular_function |
| **response to immobilization stress** | 5 | 0.0425 | biological_process |
| **programmed cell death** | 5 | 0.0451 | biological_process |
| **regulation of neuron apoptotic process** | 5 | 0.0474 | biological_process |

***S1B Table: GSEA output on enriched functional groups comparing retina from dorsal day 7 PT to uninjured dorsal retina.***

| **Name** | **# of Entities** | **p-value** | **Hit type** |
| --- | --- | --- | --- |
| **extracellular region** | 194 | 0.0117 | cellular_component |
| **response to drug** | 58 | 0.0450 | biological_process |
| **innate immune response** | 56 | 0.0165 | biological_process |
| **miRNAs** | 55 | 0.0000 | Pathway Studio Ontology |
| **immune response** | 49 | 0.0366 | biological_process |
| **proteinaceous extracellular matrix** | 34 | 0.0008 | cellular_component |
| **extracellular matrix** | 33 | 0.0001 | cellular_component |
| **intracellular membrane-bounded organelle** | 33 | 0.0287 | cellular_component |
| **response to hypoxia** | 27 | 0.0122 | biological_process |
| **extracellular matrix organization** | 25 | 0.0052 | biological_process |
| **aging** | 24 | 0.0248 | biological_process |
| **external side of plasma membrane** | 23 | 0.0111 | cellular_component |
| **receptor activity** | 21 | 0.0196 | molecular_function |
| **response to organic substance** | 20 | 0.0294 | biological_process |
| **female pregnancy** | 19 | 0.0199 | biological_process |
| **response to virus** | 18 | 0.0416 | biological_process |
| **xenobiotic metabolic process** | 17 | 0.0126 | biological_process |
| **actin cytoskeleton** | 16 | 0.0235 | cellular_component |
| **leukocyte migration** | 15 | 0.0177 | biological_process |
| **ruffle** | 13 | 0.0027 | cellular_component |
| **glutathione metabolic process** | 13 | 0.0204 | biological_process |
| **response to toxic substance** | 13 | 0.0355 | biological_process |
| **positive regulation of peptidyl-tyrosine phosphorylation** | 13 | 0.0407 | biological_process |
| **response to wounding** | 13 | 0.0425 | biological_process |
| **ubiquitin protein ligase binding** | 13 | 0.0496 | molecular_function |
| **response to nutrient** | 12 | 0.0059 | biological_process |
| **lysosomal lumen** | 12 | 0.0064 | cellular_component |
| **protease binding** | 12 | 0.0180 | molecular_function |
| **glycosaminoglycan metabolic process** | 12 | 0.0434 | biological_process |
| **negative regulation of endopeptidase activity** | 12 | 0.0462 | biological_process |
| **glutathione transferase activity** | 11 | 0.0068 | molecular_function |
| **regulation of immune response** | 11 | 0.0183 | biological_process |
| **Neuromodulator** | 11 | 0.0269 | Pathway Studio Ontology |
| **sarcolemma** | 10 | 0.0061 | cellular_component |
| **regulation of cell growth** | 10 | 0.0076 | biological_process |
| **cellular response to hypoxia** | 9 | 0.0136 | biological_process |
| **glutathione derivative biosynthetic process** | 9 | 0.0137 | biological_process |
| **positive regulation of neuron apoptotic process** | 9 | 0.0138 | biological_process |
| **neuropeptide hormone activity** | 9 | 0.0193 | molecular_function |
| **scavenger receptor activity** | 9 | 0.0467 | molecular_function |
| **cellular defense response** | 8 | 0.0027 | biological_process |
| **basement membrane** | 8 | 0.0241 | cellular_component |
| **actin filament binding** | 8 | 0.0293 | molecular_function |
| **cellular response to tumor necrosis factor** | 8 | 0.0425 | biological_process |
| **extracellular matrix structural constituent** | 7 | 0.0002 | molecular_function |
| **complement activation, classical pathway** | 7 | 0.0172 | biological_process |
| **response to nutrient levels** | 7 | 0.0242 | biological_process |
| **microvillus** | 7 | 0.0405 | cellular_component |
| **cellular response to drug** | 6 | 0.0014 | biological_process |
| **lens development in camera-type eye** | 6 | 0.0062 | biological_process |
| **platelet alpha granule lumen** | 6 | 0.0076 | cellular_component |
| **response to metal ion** | 6 | 0.0151 | biological_process |
| **collagen binding** | 6 | 0.0176 | molecular_function |
| **negative regulation of NF-kappaB transcription factor activity** | 6 | 0.0193 | biological_process |
| **glutathione peroxidase activity** | 6 | 0.0226 | molecular_function |
| **cellular response to interleukin-1** | 6 | 0.0283 | biological_process |
| **protein homotetramerization** | 6 | 0.0483 | biological_process |
| **protein binding, bridging** | 5 | 0.0011 | molecular_function |
| **protein self-association** | 5 | 0.0022 | molecular_function |
| **structural constituent of eye lens** | 5 | 0.0031 | molecular_function |
| **perikaryon** | 5 | 0.0169 | cellular_component |
| **positive regulation of cellular component movement** | 5 | 0.0177 | biological_process |
| **cytoplasmic microtubule** | 5 | 0.0332 | cellular_component |
| **biomineral tissue development** | 5 | 0.0360 | biological_process |
| **ruffle membrane** | 5 | 0.0369 | cellular_component |
| **positive regulation of intrinsic apoptotic signaling pathway** | 5 | 0.0402 | biological_process |
| **cellular response to glucocorticoid stimulus** | 5 | 0.0428 | biological_process |

***S1C Table: GSEA output on enriched functional groups comparing retina from ventral day 1 PT to uninjured ventral retina.***

| **Name** | **# of Entities** | **p-value** | **Hit type** |
| --- | --- | --- | --- |
| **cytosol** | 16 | 0.0183 | cellular_component |
| **protein heterodimerization activity** | 5 | 0.0062 | molecular_function |

***S1D Table: GSEA output on enriched functional groups comparing retina from ventral day 7 PT to uninjured ventral retina.***

| **Name** | **# of Entities** | **p-value** | **Hit type** |
| --- | --- | --- | --- |
| **miRNAs** | 17 | 0.0004 | Pathway Studio Ontology |
| **transferase activity** | 12 | 0.0126 | molecular_function |
| **signal transducer activity** | 12 | 0.0213 | molecular_function |
| **G-protein coupled receptor signaling pathway** | 10 | 0.0393 | biological_process |
| **apoptotic process** | 7 | 0.0450 | biological_process |
| **extracellular matrix organization** | 6 | 0.0199 | biological_process |
| **response to estrogen** | 5 | 0.0478 | biological_process |
